# A Functional InDel in the WRKY10 Promoter Controls the Degree of Flesh Red Pigmentation in Apple

**DOI:** 10.1002/advs.202400998

**Published:** 2024-06-14

**Authors:** Nan Wang, Wenjun Liu, Zhuoxin Mei, Shuhui Zhang, Qi Zou, Lei Yu, Shenghui Jiang, Hongcheng Fang, Zongying Zhang, Zijing Chen, Shujing Wu, Lailiang Cheng, Xuesen Chen

**Affiliations:** ^1^ Collaborative Innovation Center of Fruit & Vegetable Quality and Efficient Production, College of Horticulture Science and Engineering Shandong Agricultural University Tai'an Shandong 271000 China; ^2^ Section of Horticulture, School of Integrative Plant Science Cornell University Ithaca NY 14853 USA; ^3^ Engineering Laboratory of Genetic Improvement of Horticultural Crops of Shandong Province, College of Horticulture Qingdao Agricultural University Qingdao 266109 China; ^4^ State Forestry and Grassland Administration Key Laboratory of Silviculture in the Downstream Areas of the Yellow River, College of Forestry Shandong Agricultural University Tai'an Shandong 271000 China

**Keywords:** anthocyanin, functional InDel, red‐fleshed apple, structural variation, WRKY

## Abstract

MYB transcription factors have been linked to anthocyanin synthesis and various color phenotypes in plants. In apple, MYB10 confers a red‐flesh phenotype due to a minisatellite insertion in its R_6_ promoter, but R_6_:MYB10 genotypes exhibit various degrees of red pigmentation in the flesh, suggesting the involvement of other genetic factors. Here, it is shown that MdWRKY10, a transcription factor identified via DNA pull‐down trapping, binds to the promoter of *MdMYB10* and activates its transcription. MdWRKY10 specifically interacts with the WDR protein MdTTG1 to join the apple MYB‐bHLH‐WDR (MBW) complex, which significantly enhances its transcriptional activation activity. A 163‐bp InDel detected in the promoter region of the alleles of *MdWRKY10* in a hybrid population of identical heterozygous genotypes regarding R_6_ by structural variation analysis, contains a typical W‐box element that MdWRKY10 binds to for transactivation. This leads to increased transcript levels of *MdWRKY10* and *MdMYB10* and enhanced anthocyanin synthesis in the flesh, largely accounting for the various degrees of flesh red pigmentation in the R_6_ background. These findings reveal a novel regulatory role of the WRKY‐containing protein complex in the formation of red flesh apple phenotypes and provide broader insights into the molecular mechanism governing anthocyanin synthesis in plants.

## Graphical abstract

WRKY10 activates the transcription of *MYB10*, the gene underlying the red flesh phenotype in apple. A 163‐bp InDel in the promoter of *WRKY10* enables its own binding for transactivation, elevating the expression levels of both *WRKY10* and *MYB10*. This enhances anthocyanin synthesis, largely accounting for the various degrees of flesh red pigmentation in apple.

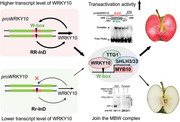

## 1 Introduction

Fruit, as a unique reproductive organ produced in the advanced stage of plant evolution, is a mutually beneficial means for plants to reproduce themselves with fruit eaters, and is only found in angiosperms and flowering plants.^[^ ^1^, ^2^ ^]^ Fruit color, which is primarily determined by chlorophylls, carotenoids, and anthocyanins, was intensively selected during the process of plant domestication and diversification for human consumption and environmental adaptation.^[^ ^3^, ^4^ ^]^ For most horticultural crops, especially those with fleshy fruits, fruit color largely determines their market value and consumer preference.^[^ ^5^, ^6^, ^7^ ^]^ In addition to the striking appearance stemming from peel color, considerable importance is increasingly given to the presence of pigments in fruit flesh, owing to their health‐promoting effects and nutritional value for plant‐derived foods.^[^ ^8^, ^9^, ^10^ ^]^ Consequently, numerous novel fruits with appealing flesh color, such as purple tomato,^[^ ^3^ ^]^ blood orange,^[^ ^11^ ^]^ and red‐fleshed apple (*Malus domestica*),^[^ ^12^ ^]^ strawberry (*Fragaria ananassa*),^[^ ^13^ ^]^ peach (*Prunus persica*),^[^ ^14^ ^]^ and kiwifruit (*Actinidia deliciosa*),^[^ ^15^ ^]^ have attracted much attention from breeders and consumers.

Anthocyanins are naturally occurring pigments ubiquitously present in the fruit of many plants that render vivid red, purple, and blue colors.^[^ ^16^, ^17^ ^]^ They are synthesized via the phenylpropanoid pathway, catalyzed by a series of enzymes encoded by structural genes.^[^ ^18^ ^]^ The expression of these structural genes are regulated by multiple transcription factor (TF) families, such as myb domain protein (MYB), basic helix‐loop‐helix (bHLH), WD repeat protein (WDR), WRKY, ethylene responsive factor (ERF), NAC domain containing protein, and basic leucine zipper.^[^ ^14^, ^19^, ^20^, ^21^ ^]^ Among them, a ternary protein complex comprising MYB, bHLH, and WDR proteins (MBW) is considered a crucial element in the regulation of anthocyanin synthesis and is well conserved in higher plants.^[^ ^22^ ^]^ MYB TFs, especially those of the two repeat (R2R3) class, are the most central TFs in the regulation of anthocyanin synthesis.^[^ ^19^ ^]^ ZmC1 in maize (*Zea mays*) was identified as the first MYB TF regulating anthocyanin biosynthesis, which led to the development of the MBW model in plants.^[^ ^23^ ^]^ Shortly afterward, the bHLH TFs ZmR and ZmB was found to be involved in anthocyanin biosynthesis in maize.^[^ ^24^ ^]^ Since then, the MBW complex that regulates anthocyanin synthesis has been extensively studied in model plants such as Arabidopsis (*Arabidopsis thaliana*),^[^ ^25^ ^]^ and petunia (*Petunia hybrida*),^[^ ^26^ ^]^ and fruit crops such as strawberry, grape (*Vitis vinifera*), apple, and pear (*Pyrus spp*).^[^ ^27^, ^28^, ^29^, ^30^ ^]^

The functional specificity of the MBW complex in regulating anthocyanin synthesis can be further modified by association with other TFs. WRKY TFs, a large TF family in plants, is well‐known to be involved in plant stress tolerance and immune responses,^[^ ^31^, ^32^ ^]^ where anthocyanins also play important roles.^[^ ^33^, ^34^ ^]^ WRKY TFs are named for the highly conserved seven amino acid sequence (WRKYGQK) at their N‐terminus, and specifically bind to the W‐box (T)TGAC(C/T) sequence.^[^ ^32^ ^]^ In Arabidopsis, a WRKY TF TRANSPARENT TESTA GLABRA2 (TTG2), interacts with the WDR protein TTG1 to regulate trichome development and proanthocyanidin synthesis.^[^ ^35^, ^36^ ^]^ In petunia, the TTG2 homolog PH3 interacts with the WDR protein AN11 to participate in vacuolar acidification and flower pigmentation.^[^ ^37^ ^]^ In apple, MdWRKY11 binds to the W‐box elements in the promoters of *MdMYB10*, *MdMYB11*, and *MdUFGT* to promote anthocyanin accumulation.^[^ ^38^ ^]^ MdWRKY40 plays an important role in wound‐induced anthocyanin biosynthesis by interacting with MdMYB1.^[^ ^39^ ^]^ MdWRKY1 binds to the promoter of the long noncoding RNA *MdLNC499*, thus inducing the expression of *MdERF109* and promoting light‐induced anthocyanin biosynthesis.^[^ ^21^ ^]^ In pear, PyWRKY26 interacts with PybHLH3 and co‐targets the *PyMYB114* promoter, thereby promoting the accumulation of anthocyanins.^[^ ^40^ ^]^ However, it is not known if any WRKY plays a role in genetic control of red phenotype in plants.

Apple is one of the most widely cultivated and economically prominent fruits in temperate regions around the world.^[^ ^41^ ^]^ However, most of the commercially important apple varieties have white or off‐white flesh with negligible anthocyanin content.^[^ ^12^ ^]^ In contrast, some apple species originating from the wild apple forests in Central Asia, such as *Malus sieversii* f. *niedzwetzkyana* have red‐flesh with high anthocyanin content, which provides precious germplasm for breeding red‐fleshed apples.^[^ ^6^, ^42^ ^]^ Compared with the R_1_ promoter allele, which contains only one repeat of a 23‐bp sequence, the R_6_ promoter allele of *MdMYB10* has a minisatellite‐like structure comprising five additional tandem repeats of the 23‐bp sequence. This R_6_ arrangement (R_6_:*MdMYB10*) allows for much stronger binding of the MdMYB10 protein to the 23‐bp sequences, forming an autoregulation loop for transactivation of *MdMYB10* expression, which leads to massive anthocyanin synthesis conferring the red‐fleshed phenotype.^[^ ^12^ ^]^ Other genetic mutations or structural variants have been reported for grape, citrus, peach, and strawberry.^[^ ^11^, ^13^, ^43^, ^44^ ^]^

In earlier work, we identified a red‐fleshed mutant being R_6_:*MdMYB10* homozygous (R_6_R_6_ genotype) that exhibits dark‐red flesh phenotype as a result of extremely high anthocyanin synthesis.^[^ ^45^ ^]^ When this mutant was crossed with *M. domestica* cv. “Gala” (R_1_R_1_ genotype), all the progenies are of R_6_R_1_ heterozygous genotype, but their fruits exhibited a wide range of flesh red pigmentation from almost no red color to completely red.^[^ ^46^ ^]^ These large variations clearly suggest genetic factors other than *MdMYB10* are involved in controlling flesh pigmentation in apple, but their identity and relationship with *MdMYB10* remains unknown.

Here, we report the identification of a WRKY family TF from apple, MdWRKY10, and its interaction with the WDR protein MdTTG1 to join the MBW complex in regulating *MYB10* expression for anthocyanin synthesis in apple flesh. Furthermore, we show that a 163‐bp InDel detected in the promoter region of alleles of *MdWRKY10* in a hybrid population of red‐fleshed apples by structural variation analysis contains a typical W‐box element that allows the binding of MdWRKY10 itself for transactivation, which largely accounts for the various degrees of red flesh phenotypes observed in the R_6_:*MdMYB10* background. These findings provide important insights into the molecular mechanism of the WRKY‐containing protein complex in the formation of red flesh apple phenotypes.

## 2 Results

### 2.1 MdWRKY10 Enhances the Transcription of *MdMYB10* by Binding to Its Promoter

**Figure 1:**
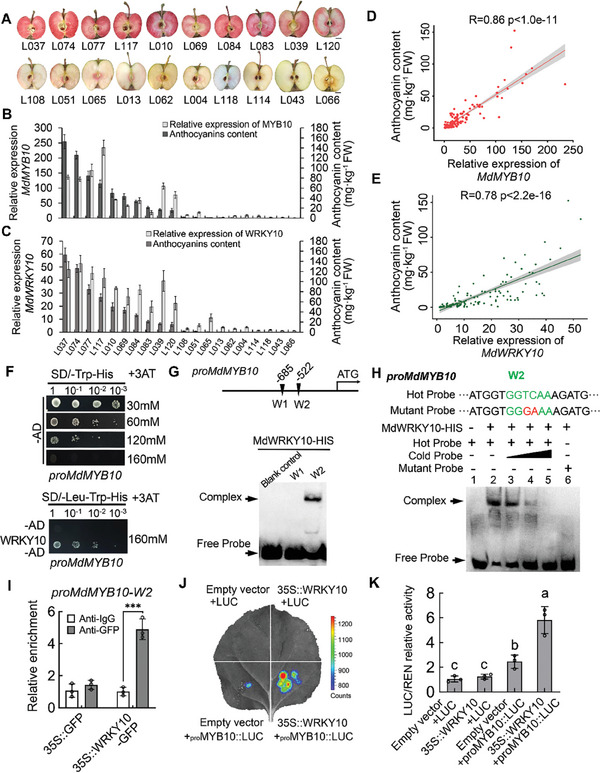
MdWRKY10 enhances the transcription of *MdMYB10* by binding to its promoter. A) Ten progenies with the typical red flesh phenotype and 10 with the non‐red flesh phenotype in the hybrid population. Scale bar = 1cm. B,C) Anthocyanin content and transcript levels of *MdMYB10* (B) and *MdWRKY10* (C) in the selected 20 progenies. D,E) Correlation analysis between anthocyanin content and transcript levels of *MdMYB10* (D) and *MdWRKY10* (E) in the flesh of all 140 progenies in the hybrid population. F) Y1H assays showing the interaction between MdWRKY10 and the promoter of *MdMYB10*. G) The W‐box cis‐elements in the promoter of *MdMYB10*. EMSA showing the binding of MdWRKY10 to the W‐box cis‐elements in the promoter of *MdMYB10*. H) EMSA showing the binding of MdWRKY10 to the W2 box in the promoter of *MdMYB10*. The hot probe was a biotin‐labeled fragment containing the W2 box. The cold probe was a non‐labeled competitive probe (at 100‐fold the concentration of the hot probe). The mutant probe contained two nucleotide mutations. I) ChIP‐qPCR assay showing binding of MdWRKY10 to the candidate W‐box elements in the promoter of *MdMYB10* in vivo. DNA fragments enriched in ChIP were used as templates for qPCR. Significant differences were determined by one‐way ANOVA followed by a Tukey's test (***P < 0.001). J) Effects of MdWRKY10 on the promoter activity of *MdMYB10* as demonstrated by a dual‐luciferase reporter assay in tobacco leaves. K) LUC/REN activity detection to verify that MdWRKY10 positively activates the transcriptional activity of *MdMYB10*. The empty LUC reporter was used as the control. Values are means ± SD of three independent biological replicates (*n* = 3). Significant differences were determined by one‐way ANOVA followed by a Tukey's test (*P* < 0.05).

To identify the genetic factor(s) that account for the variation in the degree of flesh red pigmentation, we characterized an F_1_ hybrid population of 140 progenies generated by crossing the R_6_R_6_ mutant with R_1_R_1_ “Gala” apple. They were all R_6_R_1_ heterozygous, but exhibited significantly different levels of flesh red pigmentation and anthocyanin content, ranging from almost no red color to completely red (Figures S1 and S2, Supporting Information). We compared 10 progenies exhibiting the typical red flesh phenotype with 10 progenies showing almost no red color in the flesh in terms of *MdMYB10* transcript levels and flesh anthocyanin concentrations (**Figure** **1**A). Both *MdMYB10* transcript levels and anthocyanin concentrations were significantly higher in the red‐fleshed apple fruits (Figure 1B). For the entire hybrid population, the concentration of anthocyanins in fruit flesh is highly correlated with the transcript level of *MdMYB10* (r = 0.86; Figure 1D). This close association between the *MdMYB10* expression level and the degree of flesh red coloration strongly suggests the existence of other genetic factors that regulate flesh anthocyanin level by altering the transcript level of *MdMYB10* in the same R_6_R_1_ genetic background.

To identify the upstream factors that regulate *MdMYB10* expression, the promoter sequence of *MdMYB10* was used as a probe to pull down TFs that bind to its promoter from nuclear proteins in the calli of red‐fleshed apple (R_6_R_6_) obtained earlier,^[^ ^45^ ^]^ with the proteins captured by untagged probes as a negative control (Figure S3, Supporting Information). A total of 160 proteins were identified using Liquid Chromatograph Mass Spectrometer (LC–MS), among which two unique WRKY TFs, MdWRKY10 and MdWRKY26, were selected as potential regulators for *MdMYB10* (Table S1, Supporting Information). Subsequently, we tested the transcript levels of *MdWRKY10* and *MdWRKY26* in the flesh of 10 progenies exhibiting the typical red flesh phenotype and 10 progenies showing almost no red color. Consistent with the expression pattern of *MdMYB10*, the transcript levels of *MdWRKY10* and anthocyanin concentrations were significantly higher in the red‐fleshed apple fruits (Figure 1C). In contrast, there was no significant correlation between the transcript levels of *MdWRKY26* and anthocyanin concentrations (Figure S4A, Supporting Information). For the entire hybrid population, the concentration of anthocyanins in fruit flesh is positively correlated with the transcript level of *MdWRKY10* (r = 0.78), indicating a potential function of MdWRKY10 in regulating *MdMYB10* transcription for anthocyanin synthesis (Figure 1E). Furthermore, we categorized the redness of fruit flesh in all progenies into 5 groups and then determined the transcript levels of *MdWRKY10* in each group. The results showed that an increase in the transcript levels of *MdWRKY10* is closely associated a concomitant increase in the redness of the apple fruit, further demonstrating the expression level of *WRKY10* and the degree of flesh coloration (Figure S4B, Supporting Information).

To confirm the regulatory function of MdWRKY10 in *MdMYB10* transcription, we first explored whether MdWRKY10 binds to the promoter of *MdMYB10*. A yeast one‐hybrid (Y1H) assay showed that MdWRKY10 binds to the promoter of *MdMYB10* (Figure 1F). Subsequently, analysis of the promoter of *MdMYB10* identified two W‐boxes (W1, −685; W2, −522) in its promoter (Figure 1G). We purified the MdWRKY10 protein fused to a His tag and performed electrophoretic mobility shit assays (EMSAs) to determine the binding of MdWRKY10 to these W‐boxes. MdWRKY10 caused a substantial shift of the biotin‐labeled probe that solely targets the W2 box in the *MdMYB10* promoter (Figure 1G), but the shifted band was significantly attenuated when the unlabeled probe was added as a competitor. Meanwhile, no shifted band was detected when a mutated labeled probe containing two mutated nucleotides was used (Figure 1H). Furthermore, we performed a chromatin immunoprecipitation‐quantitative real‐time PCR (ChIP‐qPCR) assay to examine the *MdMYB10* promoter regions containing W‐boxes and their enrichment levels in the DNA co‐immunoprecipitated with the green fluorescent protein (GFP) antibody relative to a non‐specific IgG antibody. MdWRKY10 substantially enhanced the enrichment levels of the *MdMYB10* promotor region containing W2 box, while having no effect on the W1 box region (Figure 1I; Figure S5, Supporting Information). These results demonstrate that MdWRKY10 binds to the second W‐box in the *MdMYB10* promoter.

Next, we investigated the effect of MdWRKY10 on the promoter activity of *MdMYB10* using a transient luciferase (LUC) assay in *N. benthamiana* leaves. The promoter sequence of *MdMYB10* was inserted into the pGreenII 0800‐LUC vector, which was fused to a *LUC* reporter gene. The coding sequence (CDS) of *MdWRKY10* was fused to the pGreenII 62‐SK vector as an effector. Co‐expression of *35S::MdWRKY10* with *proMdMYB10::LUC* led to a higher luminescence intensity compared with co‐expression of *proMdMYB10::LUC* with the empty vector control (Figure 1J,K). These results show that MdWRKY10 enhances the transcription of *MdMYB10* by binding to its promoter.

### 2.2 *MdWRKY10* Regulates Anthocyanin Biosynthesis in Apple Fruit and Calli

**Figure 2:**
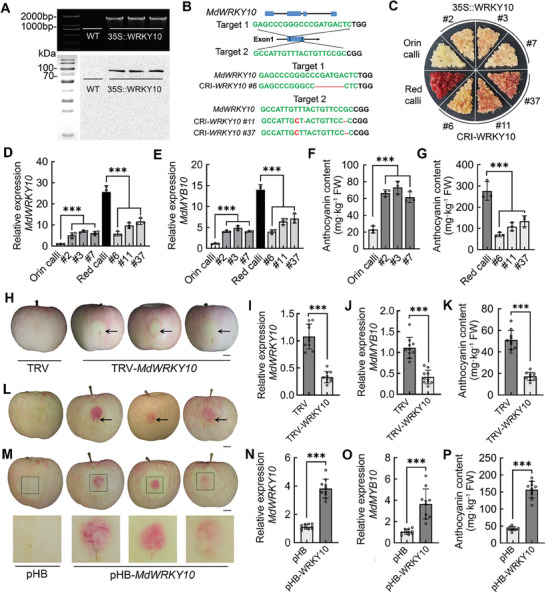
MdWRKY10 promotes anthocyanin biosynthesis in apple fruit and calli. A) The presence of the transgene in *MdWRKY10*‐overexpression calli was confirmed by PCR amplification and immunoblotting with GFP antibody. B) *MdWRKY10* knockdown calli were generated using CRISPR‐Cas9 gene editing. gRNA targeted sequences are labeled green, and the mutation or deletion sequences are labeled red. C) Wild‐type ‘Orin’ apple calli and three independent overexpression lines #2, #3, and #7; wild‐type red apple calli and three independent knockdown lines #6, #11, and #37 constructed using CRISPR/Cas9 gene editing. D,E) Transcript levels of *MdWRKY10* (D) and *MdMYB10* (E) in ‘Orin’ calli, red calli, *MdWRKY10*‐overexpression calli, and *MdWRKY10*‐knockdown calli. F,G) Anthocyanin content in *MdWRKY10*‐overexpression calli (F) and *MdWRKY10*‐knockdown calli (G). H) The phenotypes of infiltrated apple fruits with the empty TRV vector control (TRV) and three representative MdWRKY10‐knockdown repeats. Scale bar = 1 cm. I–K) Transcript levels of *MdWRKY10* (I) and *MdMYB10* (J) and anthocyanin content (K) in infiltrated apple fruits with the empty TRV vector control (TRV) and MdWRKY10‐knockdown. Values are means ± SD of independent biological replicates (*n* = 10). L) The phenotypes of infiltrated apple fruits with the empty pHB vector control (pHB) and three representative MdWRKY10‐overexpression repeats. Scale bar = 1 cm. M) Transient overexpression of *MdWRKY10* resulted in a significant increase in red color around the infiltration sites observed in apple flesh. Scale bar = 1 cm. N–P) Transcript levels of *MdWRKY10* (N) and *MdMYB10* (O) and anthocyanin content (P) in infiltrated apple fruits with the empty pHB vector control (pHB) and *MdWRKY10* overexpression. *MdActin* was used as an internal reference. Values are means ± SD of independent biological replicates (*n* = 10). Significant differences were determined by one‐way ANOVA followed by a Tukey's test (***P < 0.001).

To determine the role of *MdWRKY10* in anthocyanin biosynthesis and red pigmentation, we elevated the expression of *MdWRKY10* in apple “Orin” calli using *Agrobacterium tumefaciens*‐mediated stable transformation driven by the cauliflower mosaic virus (CaMV) 35S promoter. The transgene in *MdWRKY10*‐overexpressing (*35S::MdWRKY10*) calli was detected by both PCR and immunoblotting (**Figure** **2**A). Three independent transgenic lines #2, #3, and #7 were generated, and all three showed red pigmentation (Figure 2C). Compared with wild‐type (WT) “Orin” calli, these transgenic lines all showed higher transcript levels of *MdWRKY10* and more anthocyanin accumulation (Figure 2D,F). Meanwhile, we used the CRISPR/Cas9 gene editing system to knock down *MdWRKY10* in the calli of red‐fleshed apple (R_6_R_6_) obtained earlier.^[^ ^45^ ^]^ We designed two gRNAs targeting the first exon sequence of *MdWRKY10* based on the protospacer adjacent motif (PAM) and obtained three independent *MdWRKY10* knockdown lines, *CRI‐MdWRKY10* #6 (Target 1), and #11 and #37 (Target 2) with significant reductions in red pigmentation (Figure 2B). As shown by PCR and sequencing, all three knockdown lines displayed mutation and/or deletion sequences in the *MdWRKY10* gene, including one homozygous mutation (#6) and two heterozygous mutations (#11 and #37), all of which resulted in the generation of early ectopic stop codons (Figure S6, Supporting Information). Compared with WT red apple calli, the *MdWRKY10* expression and anthocyanin content were significantly reduced in the three knockdown lines (Figure 2D,G). Correspondingly, the *MdWRKY10*‐overexpressing calli all had higher transcript levels of *MdMYB10*, while the *MdWRKY10*‐knockdown calli all had lower transcript levels (Figure 2E). These data show that *MdWRKY10* plays an essential role in anthocyanin synthesis via transcriptional regulation of *MdMYB10*.

To elucidate the biological function of *MdWRKY10* in apple fruit, we conducted transient expression assays by infiltration of *A. tumefaciens* harboring a tobacco rattle virus (TRV) derived vector *TRV‐MdWRKY10* or *35S::MdWRKY10* vector for gene silencing and overexpression, respectively. Compared to the TRV control, suppression of *MdWRKY10* expression decreased transcript levels of *MdMYB10*, resulting in lower anthocyanin levels and attenuated red fruit coloration around the infiltrating sites (Figure 2H–K). In contrast, overexpression of *MdWRKY10* increased transcript levels of *MdMYB10* (Figure 2O), leading to significant accumulation of anthocyanins (Figure 2P) and increased fruit red coloration around the infiltrating sites (Figure 2L), which was even visible in the flesh (Figure 2M). These results suggest a positive regulatory role of *MdWRKY10* in anthocyanin accumulation and fruit red coloration.

### 2.3 MdWRKY10 Interacts with MdTTG1, but not MdMYB10, MdbHLH3, or MdbHLH33

Previous studies have shown that WDR protein TTG1 in the MBW complex interacts with other proteins such as TTG2, a WRKY TF in Arabidopsis and petunia.^[^ ^37^, ^47^ ^]^ To investigate whether *MdWRKY10* has a phylogenetic relationship and functional similarity with *TTG2*, a detailed phylogenetic analysis of the homologous genes of *MdWRKY10* and *TTG2* from additional species was performed. The phylogenetic tree showed that *MdWRKY10* and *TTG2* clearly fall into different clades (Figure S7, Supporting Information). Amino acid sequence alignment analysis indicated that *MdWRKY10* belongs to Subgroup I (SG I),^[^ ^48^ ^]^ in which all members have two WRKY domains, each followed by a C2H2 zinc finger motif separated by 16 amino acids (Figure S8, Supporting Information).

**Figure 3:**
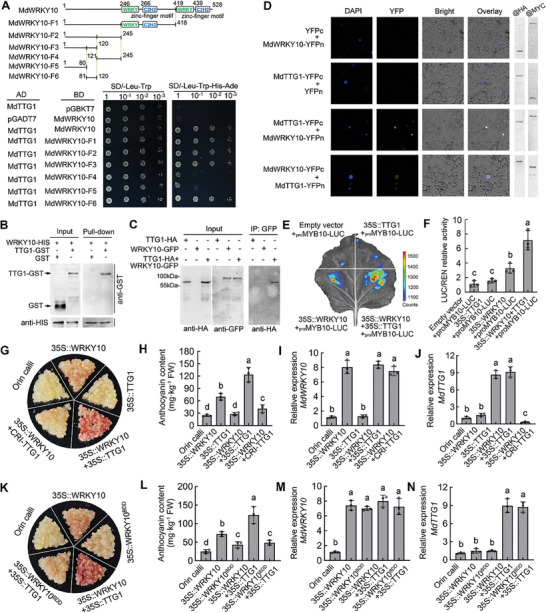
MdWRKY10 interacts with MdTTG1 in vitro and in vivo. A) Y2H assays showing the interaction of MdWRKY10 with MdTTG1. The full‐length CDS of MdWRKY10 was divided into six fragments (F1–F6); WRKY: the conserved WRKY motif; C2H2: a typical C2H2 zinc finger motif containing the CX_4_CX_22_HXH sequence; B) Pull‐down assay showing the interaction of MdWRKY10 with MdTTG1. Immunoblotting with a GST antibody showing that MdTTG1 was pulled down by MdWRKY10‐HIS. C) Co‐immunoprecipitation detection of the interaction between MdWRKY10 and MdTTG1 in vivo. The transgenic apple calli over‐expressing both MdWRKY10‐GFP and MdTTG1‐HA were used for Co‐IP assays and incubated with GFP antibody. D) BiFC assay showing the interaction of MdWRKY10 with MdTTG1. DAPI staining was used to visualize the nuclei; The right gels show the expression of the fusion protein examined by immunoblotting with anti‐HA and anti‐MYC; Scale bar = 10 µm. E) Dual‐luciferase reporter assay showing the transcriptional activation of MdWRKY10 to the *MdMYB10* promoter with the addition of MdTTG1. F) LUC/REN activity detection to verify that MdWRKY10 and MdTTG1 positively activated the transcriptional activity of *MdMYB10*. G) The phenotypes of wild‐type ‘Orin’ calli and transgenic calli, which were transformed with *35S::MdWRKY10*, *35S::MdTTG1*, *35S::MdWRKY10*+*35S::MdTTG1*, and *35S::MdWRKY10*+*CRI::MdTTG1*. H) Anthocyanin content in wild‐type and transgenic calli. I,J) Transcript levels of *MdWRKY10* (I) and *MdTTG1* (J) in wild‐type and transgenic calli. K) The phenotypes of wild‐type ‘Orin’ calli and transgenic calli, which were transformed with *35S::MdWRKY10*, *35S::MdWRKY10* ^BDD^, *35S::MdWRKY10*+*35S::MdTTG1*, and *35S::MdWRKY10* ^BDD^+*35S::MdTTG1*. L) Anthocyanin content in wild‐type and transgenic calli. M,N) Transcript levels of MdWRKY10 (M) and MdTTG1 (N) in wild‐type and transgenic calli. *MdActin* was used as an internal reference. Values are means ± SD of three independent biological replicates (*n* = 3). Significant differences were determined by one‐way ANOVA followed by a Tukey's test (*P* < 0.05).

To verify the interaction between MdWRKY10 and MdTTG1, yeast two‐hybrid (Y2H) assays were performed. We divided MdWRKY10 into six fragments (F1–F6) and found that F1–3 and F6 interacted with MdTTG1, while F4 and F5 did not, suggesting that the binding domain to MdTTG1 is located in the non‐conserved C‐terminal F6 fragment, independent of the conserved N‐terminal WRKY and C2H2 domains (**Figure** **3**A; Figure S8, Supporting Information). We also tested eight other apple SG I WRKY members by Y2H assays, but none of them interacted with MdTTG1, including the TTG2 homolog in apple, MdWRKY44, which is consistent with the non‐conservation of the MdWRKY10‐F6 fragment (Figure S9A, Supporting Information). Because MdWRKY10 is not homologous to TTG2, we then explored the conservation of the WRKY10‐TTG1 complex in model plants such as Arabidopsis and tobacco, and fruit crops such as grape and pear. We found that the WRKYs that interacted with corresponding TTG1 proteins in these species fall within the clusters of homologous genes of both TTG2 and WRKY10, but which WRKY cluster member interacts with TTG1 is not conserved across these species (Figure S9B, Supporting Information).

We then tested the interaction of MdWRKY10 with MdTTG1 in vitro using pull‐down assays. Reconstructed glutathione‐S‐transferase (GST) fusion proteins, MdTTG1‐GST, were pulled down by MdWRKY10‐His (Figure 3B). To confirm their interaction in vivo, we performed co‐immunoprecipitation (Co‐IP) assays using the transgenic apple calli overexpressing both *MdWRKY10* fused to a GFP tag and *MdTTG1* fused to a hemagglutinin (HA) tag. MdTTG1‐HA proteins in the calli were immunoprecipitated by MdWRKY10‐GFP, demonstrating their interaction (Figure 3C). The interaction was also confirmed using bimolecular fluorescence complementation (BiFC) assays in *Nicotiana benthamiana* leaves. Strong yellow fluorescent protein (YFP) signals were detected in the nucleus when *MdWRKY10* and *MdTTG1* were co‐expressed, which further indicates that MdWRKY10 interacts with MdTTG1 (Figure 3D). Next, a transient LUC assay was performed to investigate the effect of the interaction between MdWRKY10 and MdTTG1 on the promoter activity of *MdMYB10*. Co‐expression of both *35S::MdWRKY10* and *35S::MdTTG1* with *proMdMYB10::LUC* resulted in even stronger luminescence than the co‐expression of *35S::MdWRKY10* with *proMdMYB10::LUC* (Figure 3E,F), indicating that the transcriptional activation of *MdMYB10* by MdWRKY10 is further enhanced by MdTTG1.

As MdMYB10 and its partners MdbHLH3 and MdbHLH33 play important regulatory roles in anthocyanin synthesis and red flesh phenotype in apple,^[^ ^12^ ^]^ we investigated whether MdWRKY10 interacts with any of them. Y2H assays showed that MdWRKY10 did not interact with MdMYB10, MdbHLH3, or MdbHLH33 (Figure S10A, Supporting Information). This lack of interaction was confirmed by BiFC assays in *N. benthamiana leaves* (Figure S10B, Supporting Information) and pull‐down assays (Figure S10C, Supporting Information).

### 2.4 MdWRKY10's Promoting Effects on Anthocyanin Synthesis are Enhanced by Its Interaction with MdTTG1

As MdWRKY10 interacts with MdTTG1, we explored whether the promotion of anthocyanin synthesis by MdWRKY10 is dependent on MdTTG1 as a mediator. *MdWRKY10* and *MdTTG1* were separately or jointly transformed into “Orin” apple calli driven by the CaMV 35S promoter, as confirmed via both PCR amplification and immunoblotting (Figure S11A, Supporting Information). Compared with WT “Orin” calli, the *35S::MdWRKY10* transgenic lines had more anthocyanin accumulation, while no difference in anthocyanin accumulation was detected between the *35S::MdTTG1* transgenic lines and the WT control (Figure 3G). However, when *MdWRKY10* was co‐overexpressed with *MdTTG1*, the anthocyanin content in the transgenic calli was increased to a significantly higher level than that of overexpression of *MdWRKY10* alone (Figure 3H), indicating that MdTTG1 enhances the promotion effect on anthocyanin synthesis by MdWRKY10. We further generated *MdTTG1* knockdown calli by CRISPR/Cas9 in the background of *35S::MdWRKY10*‐overexpressing calli (*35S::MdWRKY10+CRI‐MdTTG1*; Figure S11B,C, Supporting Information), and found that the *MdTTG1* knockdown significantly reduced the anthocyanin content, but the knockdown calli still had higher anthocyanin levels than the WT “Orin” calli (Figure 3H). Overexpression of *MdTTG1* did not alter the transcript level of *MdWRKY10* (Figure 3I), and overexpression of *MdWRKY10* had no effect on the transcript level of *MdTTG1* either (Figure 3J), suggesting no transcriptional relationship between *MdWRKY10* and *MdTTG1*.

Furthermore, we artificially deleted the MdTTG1‐binding domain (F6 fragment) of MdWRKY10 to verify the function of MdWRKY10 after interrupting its interaction with MdTTG1 (Figure S12A, Supporting Information). Full‐length *MdWRKY10* (*35S::MdWRKY10*) and the MdTTG1 binding domain‐deleted *MdWRKY10* (*35S::MdWRKY10^BDD^*) were transformed into “Orin” apple calli, respectively. We also generated transgenic calli overexpressing *35S::MdTTG1* in the background of *35S::MdWRKY10* and *35S::MdWRKY10^BDD^* calli, as confirmed by both PCR and immunoblotting (Figure S12B, Supporting Information). The transgenic calli overexpressing *35S::MdWRKY10^BDD^* had significantly lower anthocyanin content than *35S::MdWRKY10*‐overexpressing calli (Figure 3K). Similarly, the anthocyanin level was significantly lower in the calli co‐overexpressing *35S::MdTTG1* and *35S::MdWRKY10^BDD^* than in the calli co‐overexpressing *35S::MdTTG1* and *35S::MdWRKY10* (Figure 3L). However, RT‐qPCR analysis showed no significant difference in the transcript levels of *MdWRKY10* and *MdTTG1* between the *35S::MdWRKY10* and *35S::MdWRKY10^BDD^* overexpressing calli, indicating that the decrease of anthocyanins in the *35S::MdWRKY10^BDD^* calli was caused by the lack of its interaction with MdTTG1 (Figure 3M,N). These results reveal that MdWRKY10 promotes anthocyanin synthesis in apple and this process is significantly enhanced by interaction with the mediator MdTTG1.

### 2.5 A 163‐bp InDel in the *MdWRKY10* Promoter is Associated with the Red Flesh Phenotype

**Figure 4:**
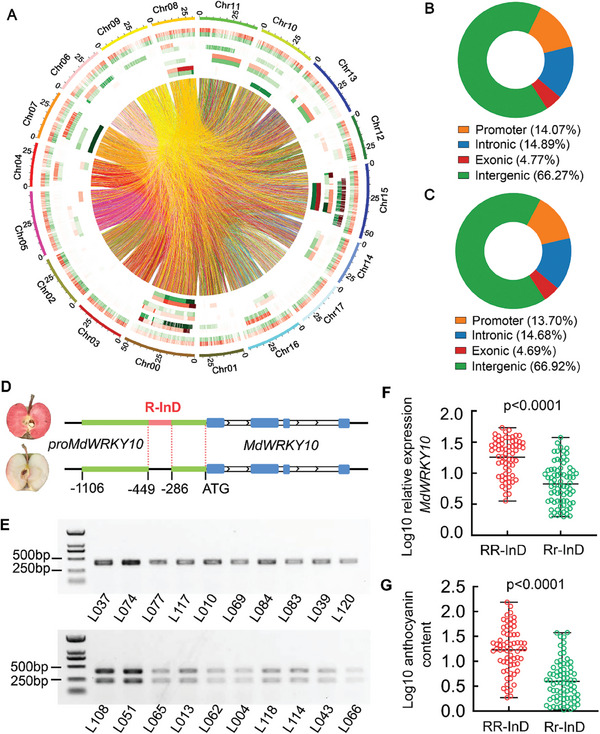
A long InDel in the *MdWRKY10* promoter is associated with the red flesh phenotype. A) Circos plot of the selective sweeps for the red‐ and non‐red‐fleshed apple progenies. The outer track represents the 17 chromosomes of the apple genome. The four inner tracks depict the structural variation distribution of insertions (INS), deletions (DEL), inversions (INV), and duplications (DUP) across the apple genome (from the outside inward). Red bars represent red‐fleshed apples. Green bars represent non‐red‐fleshed apples. B,C) The putative functional impact of the detected SVs between red‐ (B) and non‐red‐fleshed apple genomes (C). D) Molecular structure of *MdWRKY10* alleles with flanking sequences between red‐ and white‐fleshed apples. The long insertion sites in the promoter region (−449 to −286) of *MdWRKY10* are indicated by a red line (*R‐InD*). E) PCR‐based screening showing the absence or presence of the *R‐InD* insertion in the promoter of *MdWRKY10*. All red‐fleshed apple lines tested contained a long InDel‐containing band, indicating a homozygous type (*RR‐InD*), while white‐fleshed apple lines contained both a long InDel‐containing band and a short InDel‐deficient band, indicating a heterozygous type (*Rr‐InD*). F,G) Boxplots of distribution of the *MdWRKY10* expression levels (F) and the anthocyanin content (G) in the flesh of 140 apple progenies harboring the RR‐InD::MdWRKY10 or Rr‐InD::MdWRKY10 alleles. Median values are shown by horizontal lines within boxes. The *P* value is calculated based on two‐tailed Student's *t* tests.

To identify the underlying cause for the differential expression of *MdWRKY10* in the hybrid population, we analyzed structural variations (SVs) between red‐ and non‐red‐fleshed apple genomes in light of recent findings that SVs control traits such as fruit weight and shape by altering gene expression.^[^ ^44^, ^49^ ^]^ Genomic DNA of red‐ and non‐red‐fleshed apples was isolated from apple leaves and the library was constructed and sequenced on an ONT PromethION machine. SVs were called on both alignments with snifes v1.0.12 and SVIM v1.4.1. A minimum of two supporting reads were required to call an SV. The annotation of the SV was conducted by ANNOVAR. A final set of 125451 SVs were identified between the red‐ and non‐red‐fleshed apple genomes, including 64340 insertions, 60752 deletions, 101 duplications, and 258 inversions (**Figure** **4**A; Table S2, Supporting Information). The putative functional impact of these SVs was examined, and differences in both genic and intergenic regions between the red‐ and non‐red‐fleshed apple genomes were detected. Among them, 14.07% of the SVs in red‐fleshed apple genomes are located in promoter regions, compared with 13.7% of the SVs in non‐red‐fleshed apple genomes (Figure 4B,C). We found no SVs in either the exons or introns of *MdWRKY10*, but detected a 163‐bp long InDel, designated *R‐InD*, in its promoter region (−449 to −286) in the red‐fleshed but not in the non‐red‐fleshed apple genomes (Figure 4D; Figure S13, Supporting Information).

We subsequently designed primers upstream and downstream of this InDel (Table S3, Supporting Information) and verified its relationship with the red flesh phenotype by PCR. All red‐fleshed apple progenies tested displayed a long InDel‐containing band, indicating a homozygous type (*RR‐InD*), while less‐red‐fleshed apple progenies contained both a long InDel‐containing band and a short InDel‐deficient band, indicating a heterozygous type (*Rr‐InD*; Figure 4E). Moreover, the *R‐InD* region was further PCR‐amplified from all 140 progenies in the hybrid population (Figure S14, Supporting Information). The average *MdWRKY10* expression and anthocyanin content of the *RR‐InD* progenies were 21.06 and 26.96 mg kg^−1^ respectively (n = 65), which were significantly higher than that of the *Rr‐InD* progenies, 7.96 and 5.44 mg kg^−1^ (n = 75; Figure 4F,G; Table S4, Supporting Information), suggesting that the *R‐InD* may largely determine the transcript level of *MdWRKY10* and the anthocyanin content of fruit flesh. We also tested whether this *R‐InD* affects the skin color phenotype in apple. To this end, five red‐skinned and five non‐red‐skinned apple cultivars were analyzed by a specific *R‐InD* PCR marker. Both red‐skinned and non‐red‐skinned apples are *Rr‐InD* heterozygous (Figure S15, Supporting Information), suggesting that the red skin phenotype in apple is independent of *R‐InD*.

### 2.6 MdWRKY10 Directly Binds to the *R‐InD* and Transactivates Its Own Transcriptional Activity

**Figure 5:**
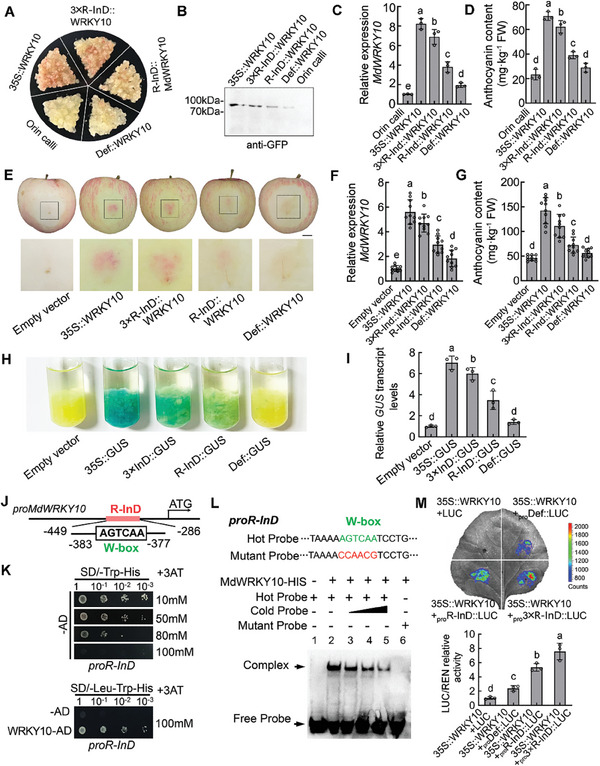
Functional analysis of the *R‐InD* sequence on the transactivation of the *MdWRKY10* promoter. A) The phenotypes of wild‐type ‘Orin’ calli and transgenic calli, which were transformed with *MdWRKY10* driven by the CaMV 35S promoter (*35S::MdWRKY10*), the *MdWRKY10* promoter containing a 3‐repeat *R‐InD* sequence (*3 × R‐InD::MdWRKY10*), *R‐InD* sequence (*R‐InD::MdWRKY10*), or *R‐InD*‐deficient sequence (*Def::MdWRKY10*). B) Presence of transgenes in transgenic calli were confirmed by immunoblotting with GFP antibody. C) Transcript levels of *MdWRKY10* in wild‐type and transgenic calli. D) Anthocyanin content in wild‐type and transgenic calli. E) The phenotypes of infiltrated apple flesh, which were transiently transformed with the empty vector, *35S::MdWRKY10*, *3 × R‐InD::MdWRKY10*, *R‐InD::MdWRKY10*, or *Def::MdWRKY10*. Scale bar = 1 cm. F) Transcript levels of *MdWRKY10* in transient transformed apple fruits. G) Anthocyanin content in transient transformed apple fruits. Values are means ± SD of independent biological replicates (*n* = 10). H) Functional analysis of *MdWRKY10* promoter activities with or without the *R‐InD* sequence using the GUS reporter gene. I) Transcript levels of the *GUS* gene in transformed apple calli. Values are means ± SD of three independent biological replicates (*n* = 3). J) A typical W‐box (AGTCAA) in the *R‐InD* sequence of the *MdWRKY10* promoter. K) Y1H assays showing the interaction between MdWRKY10 and its own promoter containing the *R‐InD* sequence. L) EMSA showing the binding of MdWRKY10 to the W‐box cis‐acting element in the *R‐InD* sequence. The hot probe was a biotin‐labeled fragment. The cold probe was a non‐labeled competitive probe. The mutant probe contained six nucleotide mutations. M) Effects of MdWRKY10 on promoter activity of *Def::LUC*, *R‐InD::LUC*, and *3 × R‐InD::LUC* as demonstrated by a dual‐luciferase reporter assay in tobacco leaves. Values are means ± SD of three independent biological replicates (*n* = 3). Significant differences were determined by one‐way ANOVA followed by a Tukey's test (*P* < 0.05).

To determine if the *R‐InD* sequence transactivates the *MdWRKY10* promoter in apple, we transformed “Orin” apple calli with *MdWRKY10* driven by promoters with an *R‐InD*‐containing sequence (*R‐InD::MdWRKY10*) or an *R‐InD*‐deficient sequence (*Def::MdWRKY10*) fused with a minimal 35S sequence. To amplify the function of *R‐InD*, we also artificially synthesized a promoter sequence containing 3‐repeats of *R‐InD* (*3 × R‐InD::MdWRKY10*), with the CaMV 35S promoter (*35S::MdWRKY10*) as a positive control (**Figure** **5**A). By targeting the GFP tag fused to MdWRKY10, we detected the expression level of MdWRKY10 protein by immunoblotting with a GFP antibody (Figure 5B). All the *R‐InD* promoters drove the expression of *MdWRKY10* to significantly higher levels than *Def::MdWRKY10*, indicating that *R‐InD* confers higher transactivation to the *MdWRKY10* promoter than the *R‐InD*‐deficient sequence (Figure 5B,C). Consistently, the anthocyanin content in the *3 × R‐InD::MdWRKY10* and *R‐InD::MdWRKY10* transgenic calli was significantly higher than that in the *Def::MdWRKY10* or WT “Orin” calli, but did not reach the level of *35S::MdWRKY10* (Figure 5D). To verify the transactivation effect of *R‐InD* in apple fruit, we transiently expressed *35S::MdWRKY10*, *3 × R‐InD::MdWRKY10*, *R‐InD::MdWRKY10*, and *Def::MdWRKY10* via *A. tumefaciens* infiltration (Figure 5E). The expression of *MdWRKY10* in *35S::MdWRKY10*, *3 × R‐InD::MdWRKY10*, and *R‐InD::MdWRKY10* was significantly higher than that of *Def::MdWRKY10* or the empty vector control (Figure 5F), resulting in significantly higher red fruit coloration and anthocyanin accumulation around the infiltrating sites (Figure 5G). These data confirm the transactivation function of *R‐InD* to the *MdWRKY10* promoter. We further confirmed the transactivation of *MdWRKY10* promoter by the *R‐InD* sequence using β‐glucuronidase (GUS) staining on transgenic “Orin” apple calli expressing *GUS* driven by a 3 *× R‐InD*‐containing sequence (*3 × R‐InD::GUS*), *R‐InD*‐containing sequence (*R‐InD::GUS*) or *R‐InD*‐deficient sequence (*Def::GUS*) fused with a minimal 35S sequence, with the empty promoter (*Empty::GUS*) and CaMV 35S promoter (*35S::GUS*) as negative and positive controls, respectively (Figure 5H). The *GUS* expression of *3 × R‐InD::GUS* and *R‐InD::GUS* was significantly higher than that of *Empty::GUS*, while the *GUS* expression of *Def::GUS* was not significantly different from the *Empty::GUS* (Figure 5I), confirming the transactivation effect of the *R‐InD* sequence on the *MdWRKY10* promoter activity.

To explore how *R‐InD* affects the transcriptional activity of *MdWRKY10*, we analyzed the cis‐elements in its sequence using PlantPAN 3.0. We found a typical W‐box (AGTCAA) in the *R‐InD* sequence for the recognition and binding by WRKY TFs (Figure 5J). We next determined whether MdWRKY10 binds to the *R‐InD* in its own promoter to generate an autoregulatory function, just as MdMYB10 does by binding to its own R_6_ repeat sequence.^[^ ^12^ ^]^ A Y1H assay showed that MdWRKY10 binds to the *R‐InD* (Figure 5K). To confirm the result, we purified the MdWRKY10 protein and performed EMSAs. MdWRKY10 caused a shift of the biotin‐labeled probe, indicating the presence of the protein‐DNA complex. When an unlabeled probe was added as a competitor, the binding of MdWRKY10 to the *R‐InD* was attenuated. Meanwhile, no shifted band was detected when a mutated labeled probe containing six mutated nucleotides was used (Figure 5L). These results demonstrate that MdWRKY10 binds to the W‐box in the *R‐InD*.

Next, we validated the effects of MdWRKY10 on the promoter activity of *R‐InD* using transient LUC assays in *N. benthamiana* leaves. The promoters with a 3 *× R‐InD*‐containing sequence (*3 × R‐InD::LUC*), *R‐InD*‐containing sequence (*R‐InD::LUC*), or *R‐InD*‐deficient sequence (*Def::LUC*) were inserted individually into the pGreenII 0800‐LUC vector, which was fused to a LUC reporter gene. The CDS of *MdWRKY10* was fused to the pGreenII 62‐SK vector as an effector. Luminescence detection revealed that co‐expression of *35S::MdWRKY10* with *3 × R‐InD::LUC* yielded the strongest luminescence intensity, followed by *R‐InD::LUC*, with *Def::LUC* being the weakest (Figure 5M). These results indicate that MdWRKY10 activates its own transcriptional activity by binding to *R‐InD*.

### 2.7 ChIP‐Seq Identifies Genomic Sites Bound By MdWRKY10

**Figure 6:**
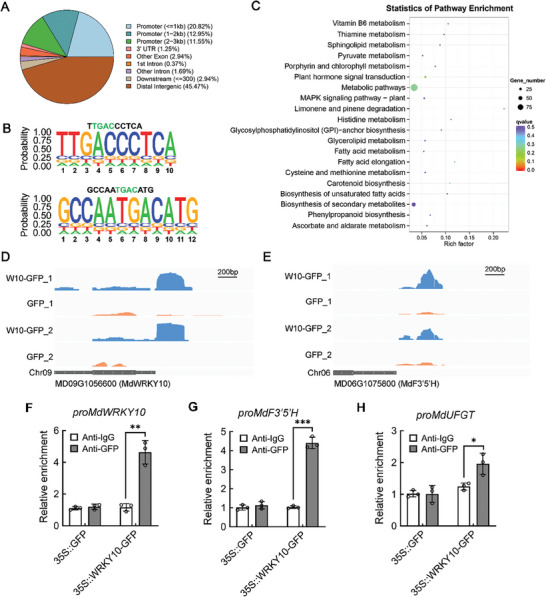
ChIP‐seq analysis to screen genomic sites that are directly bound by MdWRKY10. A) ChIP sequenced reads were mapped to the reference genome, and the peak distribution of the functional region of each gene is shown in the pie chart. B) The conserved motifs containing typical W‐box elements in the genomic regions of peak binding are listed. The W‐box sequences are labeled green. C) KEGG pathway enrichment analyses of the genes overlapping the MdWRKY10 binding peaks. The size of the dot represents the number of genes annotated to the KEGG pathways. The color from red to purple represents the significance of enrichment. D,E) MdWRKY10 occupancy at the *MdWRKY10* promoter (D) and *MdF3’5’H* promoter (E) based on global ChIP‐seq data, which were mapped onto the apple genome coordinates and visualized using the Integrative Genomics Viewer. F–I) ChIP‐qPCR assay showing binding of MdWRKY10 to the candidate W‐box elements in the promoters of (F) *MdWRKY10*, (G) *MdF3’5’H*, and (H) *MdUFGT* in vivo. DNA fragments enriched in ChIP were used as templates for qPCR. Values are means ± SD of three independent biological replicates. Significant differences were determined by one‐way ANOVA followed by a Tukey's test (*P < 0.05; **P < 0.01; ***P < 0.001).

To identify other downstream target genes of MdWRKY10 that may be involved in anthocyanin synthesis, we performed a ChIP‐seq assay using GFP antibody and chromatin extracted from GFP‐tagged *35S::MdWRKY10* apple calli, with the empty GFP‐tagged apple calli as a negative control. The DNA fragments bound to MdWRKY10‐GFP and empty‐GFP were co‐immunoprecipitated, and then released for constructing libraries in two replicates. They were sequenced on an Illumina HiSeq platform and 50‐bp single‐end reads or 150‐bp paired‐end reads were generated. In total, 1801 peaks were detected in both replicates and were used for further analyses (Table S5, Supporting Information). By analyzing the distribution of binding peaks at the transcription start site (TSS) of related genes, we found that there are significantly enriched binding peaks within 1000 bp from the TSSs (Figure S16, Supporting Information). Approximately 51.57% of the 1801 MdWRKY10 binding peaks were located in the genic regions, of which 45.32% were located in the promoter regions (20.82% < = 1 kb; 12.95% 1–2 kb; 11.55% 2–3 kb), 1.25% in the 3′‐UTRs, 2.94% in other exon regions, and 2.06% in intron regions (**Figure** **6**A). We subsequently analyzed the conserved motifs in the genomic regions of peak binding and identified most significantly conserved motifs containing 10 or 12 bases (Figure S17, Supporting Information), each of which included the core part of the W‐box cis‐element (Figure 6B).

KEGG pathway analysis on the genes overlapping the MdWRKY10 binding peaks showed that those in metabolic pathways, biosynthesis of secondary metabolites, plant hormone signal transduction, the MAPK signaling pathway, and phenylpropanoid biosynthesis are significantly enriched (Figure 6C). Among the overlapping genes, we identified eight WRKY TFs, which suggests that MdWRKY10 may have a universal regulatory effect on downstream WRKY TFs, including itself (Figure 6D). We also screened out a flavonoid 3′,5′‐hydroxylase gene, *MdF3’5’H*, and a UDP‐glucose flavonoid 3‐O‐glucosyltransferase gene, *MdUFGT*, which are two key enzymes in anthocyanin synthesis^[^ ^50^ ^]^ with significant MdWRKY10 binding peaks in their promoter regions (Figure 6E; Figure S18, Supporting Information). Furthermore, we performed a ChIP‐qPCR assay to examine the binding peaks in the promoters of *MdWRKY10*, *MdF3’5’H*, and *MdUFGT*, and their enrichment levels in the DNA co‐immunoprecipitated with the GFP antibody relative to a non‐specific IgG antibody. MdWRKY10 substantially enhanced the enrichment levels of the binding peaks in the promoters of *MdWRKY10*, *MdF3’5’H*, and *MdUFGT* (Figure 6F–I). These results suggest that MdWRKY10 binds to the promoters of *MdF3’5’H* and *MdUFGT* as well as its own promoter.

### 2.8 MdWRKY10 Enhances the Transcription of *MdF3’5’H* and *MdUFGT* by Binding to Their Promoters

**Figure 7:**
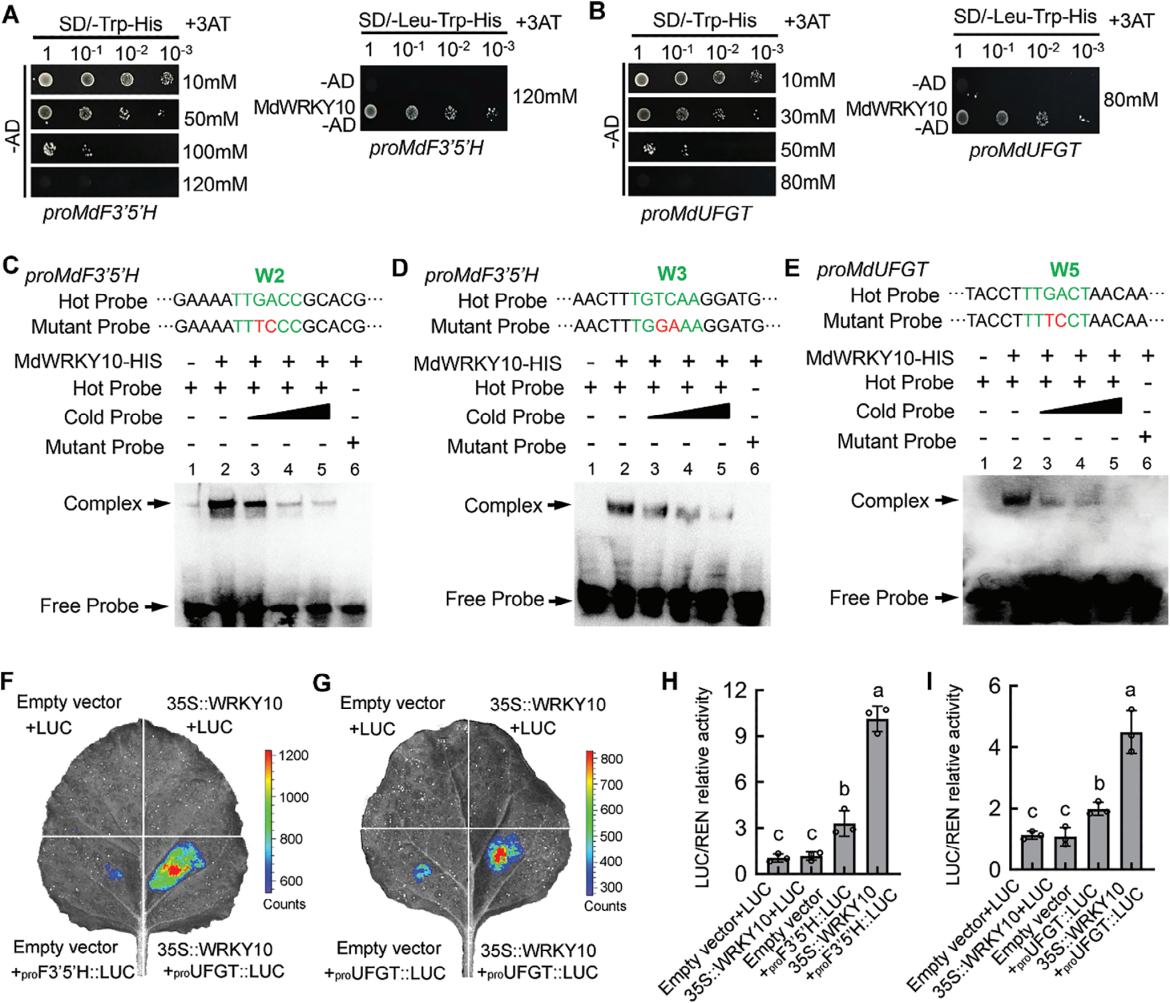
MdWRKY10 enhances the transcription of *MdF3’5’H* and *MdUFGT* by binding to their promoters. A,B) Y1H assays showing the interaction between MdWRKY10 and the promoters of *MdF3’5’H* (A) and *MdUFGT* (B). The background leakiness of the pHIS2 vectors was suppressed using 3‐amino‐1,2,4‐triazole (3‐AT). C–E) EMSA showing the binding of MdWRKY10 to the W‐box cis‐acting elements in the promoters of *MdF3’5’H* (W2 and W3) and *MdUFGT* (W5). The hot probe was a biotin‐labeled fragment containing the W‐box. The cold probe was a non‐labeled competitive probe (at 100‐fold the concentration of the hot probe). The mutant probe contained two nucleotide mutations. F,G) Effects of MdWRKY10 on the promoter activity of *MdF3’5’H* (F) and *MdUFGT* (G) as demonstrated by a dual‐luciferase reporter assay in tobacco leaves. The empty vector was used as a control. H,I) LUC/REN activity detection to verify that MdWRKY10 positively activated the transcriptional activity of *MdF3’5’H* (H) and *MdUFGT* (I) by binding to their promoters. The empty LUC reporter was used as a control. Values are means ± SD of three independent biological replicates (*n* = 3). Significant differences were determined by one‐way ANOVA followed by a Tukey's test (*P* < 0.05).

To verify the binding of MdWRKY10 to the promoters of *MdF3’5’H* and *MdUFGT*, we first conducted an Y1H assay, which showed that MdWRKY10 binds to the promoters of both *MdF3’5’H* and *MdUFGT* (**Figure** **7**A,B). Next, analysis of the promoters of *MdF3’5’H* and *MdUFGT* indicated that *MdF3’5’H* and *MdUFGT* contain three W‐boxes (W1, −269; W2, −439; W3, −1252), and two W‐boxes (W4, −1078; W5, −1380), respectively (Figure S19A, Supporting Information). We purified the MdWRKY10 protein fused to a His tag and performed EMSAs. We found that MdWRKY10 binds to the promoters of *MdF3′5′H* (W2 and W3) and *MdUFGT* (W5) (Figure S19B, Supporting Information). Addition of the unlabeled probe led to attenuation of the binding of MdWRKY10, and the binding was completely abolished with a mutated labeled probe containing two mutated nucleotides (Figure 7C–E). These results demonstrated that MdWRKY10 directly binds to the W‐boxes of *MdF3′5′H* and *MdUFGT* promoters.

We subsequently determined the effects of MdWRKY10 on the promoter activity of *MdF3’5’H* and *MdUFGT* using transient LUC assays in *N. benthamina* leaves. The promoter sequences of *MdF3’5’H* and *MdUFGT* were inserted individually into the pGreenII 0800‐LUC vector, which was fused to a *LUC* reporter gene. The CDS of *MdWRKY10* was fused to the pGreenII 62‐SK vector as an effector. Luminescence detection revealed that co‐expression of *35S::MdWRKY10* with *proMdF3’5’H::LUC* generated remarkably stronger luminescence compared with expression of *proMdF3’5’H::LUC* in combination with the empty vector controls (Figure 7F,H). Similar results were obtained for the promoter activity of *proMdUFGT::LUC* (Figure 7G,I). These results suggest that MdWRKY10 enhances the transcription of *MdF3’5’H* and *MdUFGT* by binding to their promoters for anthocyanin biosynthesis.

## 3 Discussion

Earlier work showed that *MdMYB10* plays a critical role in anthocyanin synthesis in apple fruit flesh.^[^ ^12^, ^29^ ^]^ The R_6_ promoter of *MdMYB10* with a minisatellite insertion of 5 direct tandem repeats of a 23‐bp sequence drastically enhances its expression via autoregulation by its own protein, resulting in a red flesh phenotype.^[^ ^12^ ^]^ However, presence of *R_6_:MYB10* alone does not guarantee a red flesh phenotype as demonstrated by large variations in flesh red pigmentation from almost no red color to completely red in R_6_R_1_ heterozygotes.^[^ ^46^, ^51^ ^]^ This clearly suggests that genetic factors other than *MdMYB10* are involved in controlling flesh red pigmentation in apple. Here, we found that the *MdWRKY10* gene, located on linkage group (LG) 9 of the apple genome, co‐locating with *MdMYB10*, is involved in regulating *MdMYB10* expression, which is different from a QTL peak detected earlier at the top of LG16 by linkage analysis of a hybrid population containing homozygous alleles R_1_R_1_ and R_6_R_6_.^[^ ^52^ ^]^ We show that MdWRKY10 binds to the promoter of *MdMYB10*, activating its expression for anthocyanin synthesis in apple flesh. MdWRKY10 specifically interacts with WDR protein MdTTG1 to join the MBW complex, which significantly enhances its transactivation activity. A 163‐bp InDel in the promoter region of *MdWRKY10* contains a typical W‐box element that enables the binding by its own protein for transactivation. This leads to increased transcript levels of *MdWRKY10* and *MdMYB10* and enhanced anthocyanin synthesis in the flesh, largely accounting for the variations in flesh red pigmentation in the same R_6_R_1_ genetic background. These findings reveal a key role of *MdWRKY10* in regulating the expression level of *MdMYB10* for controlling red flesh apple phenotypes, which not only brings us one step closer to gaining a complete picture of the genetic control of anthocyanin synthesis in apple flesh, but also provides broader insights into the molecular interactions that regulate anthocyanin synthesis in plants.

### 3.1 WRKY10 Transcriptionally Regulates *MYB10* As Well As *MdF3’5’H* and *MdUFGT*, Enhancing Anthocyanin Synthesis in Apple Flesh

MdWRKY10 transcriptionally regulates *MdMYB10* by binding to its promoter. Of the two W‐boxes present in the promoter of *MdMYB10*, MdWRKY10 specifically binds to the second W box (Figure 1E,F), activating its transcription as demonstrated by the luciferase reporter assay (Figure 1H). *MdWRKY10* knockdown and overexpression lead to decreased and increased levels of *MdMYB10* transcripts and anthocyanin levels in both calli and fruit, respectively (Figure 2E,J,O). These data show that MdWRKY10 plays an essential role in anthocyanin synthesis via transcriptional regulation of *MdMYB10*. In addition, MdWRKY10 also directly binds to the promoters of the enzyme genes *MdF3’5’H* and *MdUFGT* in the anthocyanin synthesis pathway, enhancing their transcript levels (Figure 7). Transcriptional regulation of these genes by MdWRKY10 underlies the close correlation between its transcript levels and fruit flesh anthocyanin concentrations detected in the entire hybrid population with the same R_6_R_1_ genetic background (Figure 1C).

Phylogenetic analysis revealed that the previously reported WRKY TFs that regulate anthocyanin synthesis are distributed in Groups I, II, and III, of which WRKY44 in Group I and WRKY75 and WRKY40 in Group II enhance whereas WRKY41 in Group III represses anthocyanin synthesis.^[^ ^39^, ^53^, ^54^, ^55^ ^]^ MdWRKY10 identified in this study also belongs to Group I, but is not in the same branch as the TTG2 (WRKY44) homologous genes (Figure S7, Supporting Information).

### 3.2 The Transcriptional Activation Activity of WRKY10 is Enhanced by Its Interaction with TTG1

The MBW ternary complex consisting of MYB, bHLH, and WDR proteins regulates anthocyanin synthesis.^[^ ^22^ ^]^ However, the complex differs between species due to changes in its participating members, and can be further modified by association with other TFs. In apple, bHLHs interact with both the MYB proteins and the WDR protein MdTTG1, forming the MBW complex.^[^ ^29^, ^56^, ^57^ ^]^ In this study, several in vitro and in vivo interaction assays showed that MdWRKY10 specifically interacts with MdTTG1 to join the apple MBW complex (Figure 3). Moreover, we found that the domain in MdWRKY10 that interacts with MdTTG1 resides in its non‐conserved C‐terminal F6 segment, independent of the conserved N‐terminal WRKY and C2H2 domains, indicating the specificity of their interaction. When *MdWRKY10* was co‐overexpressed with *MdTTG1*, the anthocyanin content in transgenic calli was significantly increased compared with overexpression of *MdWRKY10* alone. In contrast, when the interaction between MdWRKY10 and MdTTG1 was interrupted by silencing the expression of *MdTTG1* or deleting the binding domain (F6 fragment), the anthocyanin content in the transgenic calli was significantly reduced (Figure 3K–N). These results indicate that the transcriptional activation activity of MdWRKY10 is enhanced by its interaction with MdTTG1. In earlier studies, the WRKY TF TTG2 and its homologs were also found to interact with WDR proteins, forming an MBWW quaternary complex in regulating the synthesis of anthocyanins and proanthocyanidins in Arabidopsis.^[^ ^36^, ^47^ ^]^ In petunia, the WRKY TF PH3 co‐regulates vacuolar acidification and flower pigmentation with the PH4‐AN1‐AN11 complex, resulting in diverse flower color phenotypes.^[^ ^37^ ^]^ We found that MdWRKY10 interacts with MdTTG1, whereas the TTG2 homolog MdWRKY44 in apple does not, further illustrating the species‐specific interaction between WRKY TFs and the MBW complex that enriches the functional diversity of the MBW complex.

Earlier studies showed that WRKY TFs regulate plant anthocyanin synthesis by interacting with MYBs or bHLHs in addition to WDR proteins in the MBW ternary complex. In apple, MdWRKY40 interacts with MdMYB1 to enhance the binding of MdMYB1 to its target genes for anthocyanin synthesis in response to wounding.^[^ ^39^ ^]^ Similarly, SmWRKY44 interacts with SmMYB1 to jointly promote the biosynthesis of anthocyanins in eggplant leaves.^[^ ^55^ ^]^ PyWRKY26 interacts with PybHLH3 and they co‐target the *PyMYB114* promoter to regulate anthocyanin biosynthesis and transport in red‐skinned pears.^[^ ^40^ ^]^ However, MdWRKY10 does not interact with MdMYB10, MdbHLH3 or MdbHLH33 as demonstrated by both the in vitro and in vivo interaction assays (Figure S10, Supporting Information). Considering the significant spread in *MdWRKY10* expression levels within *RR‐InD* and *Rr‐InD* genotypes (Figure 4F), it is possible that other interacting partners or TFs for MdWRKY10 may further modify the function of MdWRKY10.

### 3.3 *R‐InD* Enables Binding of WRKY10 to Its Own Promoter for Transactivation, Governing the Degree of Flesh Red Pigmentation in the R_6_R_1_ Background

Structural variation analysis of apple progenies with different red flesh phenotypes identified a 163‐bp insertion (*R‐InD*) in the promoter region of *MdWRKY10* (Figure 4), with all the red‐fleshed ones being *R‐InD* homozygous while the less‐red‐fleshed ones being heterozygous in the R_6_R_1_ population (Figure S14, Supporting Information). Both stable transformation in apple calli and transient expression in apple fruit indicated that *R‐InD* confers higher transactivation of the *MdWRKY10* promoter, thereby promoting its transcriptional level and anthocyanin synthesis. GUS staining assays confirmed that the *MdWRKY10* promoter containing *R‐InD* drives a stronger GUS activity than the *R‐InD* deficient promoter (Figure 5). The *R‐InD* sequence contains a canonical W‐box that is specifically recognized and bound by MdWRKY10 to enhance its own transcription, which is analogous to the autoregulation of the *MdMYB10* gene characterized earlier in red‐fleshed apples.^[^ ^12^ ^]^ The difference in anthocyanin levels between *R‐InD* homozygous and heterozygous genotypes in the R_6_R_1_ genetic background correspond to the difference in *MdWRKY10* expression levels between the two groups (Figure 4F,G), a clear indication of gene dosage effect. This suggests that a threshold level of *MdWRKY10* expression is required for enhancing the expression level of *MdMYB10* to a high enough degree to trigger its autoregulation for significant up‐regulation of anthocyanin synthesis and accumulation in the apple flesh, and the *MdWRKY10* expression level in the *R‐InD* heterozygous genotypes is below this threshold.

Insertion of a gypsy‐like LTR retrotransposon (redTE) in the upstream of *MdMYB1* was reported to control the red color of apple peel.^[^ ^58^ ^]^ However, no redTE insertion was detected in red‐fleshed apples, suggesting independently inherited features of the red‐fleshed and red‐skinned traits.^[^ ^58^ ^]^ Our finding that both red‐skinned and non‐red‐skinned apples are *Rr‐InD* heterozygous suggests that the red skin phenotype in apple was independent of *R‐InD*.

The structural variants of genes previously identified for regulating the anthocyanin pathway are largely MYB transcription factors. In grapes, insertion of a Gret1 retrotransposon in the promoter of *VvMYBA1* suppresses its transcript level, resulting in the absence of anthocyanins and consequently a white or green phenotype for berry skin color.^[^ ^45^ ^]^ In citrus, insertion of a copia‐like LTR retrotransposon Tcs1 in the promoter of the MYB TF, *Ruby*, significantly increases its transcript levels, conferring a prominent red fruit flesh phenotype.^[^ ^11^ ^]^ In strawberry, insertion of a gypsy LTR retrotransposon in the coding region of *FaMYB10* blocks its expression, rendering a white fruit phenotype while insertion of a CACAT‐like transposon in the *FaMYB10‐2* promoter enhances its expression, leading to a red flesh phenotype.^[^ ^13^ ^]^ In peach, a 487‐bp deletion in the *PpMYB10.1* promoter enhances its transcriptional activation activity, underpinning flesh color formation around the stone.^[^ ^44^ ^]^ The only WRKY transcription factor that has structural variations characterized is *PH3* in petunia, where a *dTPH1* transposon insertion in its coding region alters the expression of genes involved in vacuolar acidification, resulting in a variety of flower pigmentation in the derived recessive *ph3* mutants and transposon‐tagged *ph3* mutants.^[^ ^37^ ^]^ Identification and functional characterization of these structural variants have made it possible to develop gene‐specific markers for marker‐assisted selection of desired plant color phenotype, which is particularly important for both fruit peel and flesh color of woody perennial fruit crops as they have a long juvenile period.^[^ ^59^, ^60^ ^]^ In terms of breeding apples for the red‐flesh trait, identification of *R‐InD* described in this work represents a significant step toward fully understanding the genetic control of anthocyanin synthesis in apple flesh since the *MdMYB10* gene was discovered.^[^ ^12^, ^29^ ^]^

**Figure 8:**
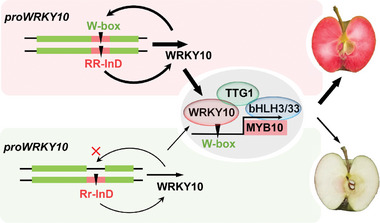
A proposed working model on the role of MdWRKY10 in regulating anthocyanin synthesis in apple flesh. The functional 163‐bp insertion (R‐InD) in the *MdWRKY10* promoter contains a typical W‐box cis‐acting element that enables binding by its own protein, thereby enhancing its transactivation activity to a higher level than the allele without the insertion (r‐InD). MdWRKY10 specifically interacts with WDR protein, MdTTG1, to join the MYB10‐bHLH3/33‐WDR (TTG1) complex. MdWRKY10 binds to the promoter of *MdMYB10* as well as those of structural genes *MdF3’5’H* and *MdUFGT* (not shown) in the anthocyanin synthesis pathway, activating their transcription to enhance anthocyanin synthesis in the apple flesh. In the homozygous RR‐InD genotype, the *WRKY10* expression level is enhanced to a significantly higher level due to the presence of two copies of the functional R‐InD compared with only one functional copy of R‐InD in the heterozygous Rr‐InD genotype. This higher *WRKY10* expression in the RR‐InD genotype is amplified via the autoregulation of *MYB10* expression, leading to a much higher level of anthocyanin synthesis consequently rendering the red flesh phenotype.

Based on the findings presented here and elsewhere, we propose a working model on the role of *MdWRKY10* in regulating anthocyanin synthesis in apple flesh (**Figure** **8**). The functional 163‐bp insertion (*R‐InD*) in the *MdWRKY10* promoter contains a typical W‐box that enables the binding by its own protein, enhancing its transactivation activity to a higher level than the allele without the insertion (*r‐InD*). The MdWRKY10 protein binds to the promoter of *MdMYB10* and two structural genes *MdF3’5’H* and *MdUFGT*, activating their expression for anthocyanin synthesis. In addition, MdWRKY10 interacts with WDR protein MdTTG1 to join the MBW complex, enhancing the transactivation activity of MdWRKY10. In the homozygous *RR‐InD* genotype, two copies of the functional *R‐InD* allele elevate the *MdWRKY10* expression to a significantly higher level than in the heterozygous *Rr‐InD* genotype. This significantly higher level of *MdWRKY10* expression is subsequently amplified via the autoregulation of *MdMYB10*, enhancing anthocyanin synthesis in apple flesh to a much higher level leading to the red flesh phenotype. These findings reveal a key role of MdWRKY10 in the genetic control of red flesh phenotype in apple and provide broader insights into the molecular interactions for the regulation of anthocyanin synthesis in plants.

## 4 Experimental Section

### Plant Materials

The F_1_ hybrid population of red‐fleshed apple progenies were grown at the Fruit Tree Breeding Base in Liaocheng, China (36°580 N, 115°420 E). They were planted in 2014 and first fruited in 2017. The 140 progenies in the population were labeled as L001 to L140. The fruit flesh of each apple progeny in the F_1_ hybrid population was collected in 2018, 2019, and 2020 in biological triplicate, with 10 fruits per replicate, and then stored at −80 °C until use.

The red calli derived from a red flesh apple mutant (R_6_R_6_) were cultured as described previously.^[^ ^61^ ^]^ The calli were cultured on a solid medium consisting of Murashige and Skoog (MS) medium supplemented with 1 mg L^−1^ 6‐benzylaminopurine (6‐BA), 0.5 mg L^−1^ α‐naphylacetic acid (NAA), 30 g L^−1^ sucrose, and 7.5 g L^−1^ agar at 24 °C under a 16‐h light/8‐h dark photoperiod (light intensity: 100±10 µmol m^−2^ s^−1^ with fluorescent lamps), and subcultured every 20 d. The “Orin” calli were cultured as described previously^[^ ^45^ ^]^ on a solid medium consisting of MS supplemented with 1 mg L^−1^ 2, 4‐dichlorophenoxyacetic acid (2, 4‐D), 0.5 mg L^−1^ 6‐BA, 30 g L^−1^ sucrose, and 7 g L^−1^ agar in the dark at 24 °C and subcultured every 20 d.

### Determination of Anthocyanin Content

Anthocyanin content was determined as described previously.^[^ ^62^ ^]^ Each sample (0.5 g) was weighed and then ground into a powder in liquid nitrogen before being extracted in 5 mL of 1% (v/v) HCl‐methanol for 24 h in the dark at 4 °C. The mixture was centrifuged at 8000 *g* for 10 min, and then 1 mL of supernatant was added to 4 mL of KCl buffer (pH 1.0, 0.025 M), and another 1 mL was added to 4 mL of NaAc buffer (pH 4.5, 0.4 M). Both mixtures were incubated at 4 °C for 15 min. Then, the absorbance of each mixture was measured at 510 nm and 700 nm using a UV2450 spectrophotometer (Shimadzu, Kyoto, Japan).

### Nuclear Protein Extraction and DNA Pull‐Down Assay

Nuclear proteins were extracted from red apple calli following the manufacturer's instructions using a Nuclear and Cytoplasmic Extraction kit (Cwbio, Jiangsu, China). Each sample (0.5 g) of red apple calli was weighed and then ground into a powder in liquid nitrogen and mixed with 200 µL of extraction buffer. The protein extraction procedure was performed three times to obtain sufficient nuclear proteins for DNA pull‐down assays. Single‐strand probes from *MdMYB10* promoter for DNA pull‐down were synthesized and labelled with biotin tags at Sangon Biotech (Sangon Biotech, Shanghai, China).

DNA pull‐down assay was performed as previously described with slight modifications.^[^ ^63^ ^]^ 200 µL of Dynabeads M‐280 Streptavidin (Invitrogen, California, US) was added into a fresh 1.5 mL tube. The microfuge tube was secured onto PolyATrace 1000 magnet (Promega, Wisconsin, US) for 1 min to pull Dynabeads down and remove the supernatant. The beads were washed with 500 µL 2 *×* B&W buffer (10 mM Tris–HCl pH 7.5, 1 mM EDTA, 2 M NaCl) for three times, and re‐suspended in 200 µL 2 × B&W buffer, followed by addition of 200 µL of biotin labeled probe. After mixing, the mixture was incubated at room temperature on a shaking table for 30 min. The beads were pulled down using the PolyATract 1000 magnet, and the supernatant removed. Repeatedly incubating beads with biotin labeled probes to ensure adsorption of saturated DNA. The probe‐beads mixture was washed with 500 µL of protein binding buffer (20 mM Tris–HCl pH 8.0, 1 mM EDTA, 10% glycerol, 100 mM NaCl, 0.05% Triton X‐100, 1 mM DTT) to ensure that the DNA probe was under reaction conditions suitable for DNA‐protein interaction. The mixture was re‐suspended in 200 µL protein binding buffer, then 400 µL of protein extraction buffer with 10 µL of Poly dI dC (ThermoFisher, Massachusetts, US) was added and rolled at 4 °C for 1 h. The bead‐probe‐protein complex was pulled down using magnet and washed with 500 µL protein binding buffer containing 10 µL Poly dI dC for five times. The captured proteins were eluted with 50 µL of elution buffer (25 mM Tris HCl pH 8.0, 100 mM NaCl), separated by sodium dodecyl sulphate polyacrylamide gel electrophoresis (SDS‐PAGE), and detected using LC–MS.

### Y2H Assays

Y2H assays were performed to determine the interaction between MdWRKY10 and MdTTG1, MdMYB10, MdbHLH3, and MdbHLH33. The CDS of *MdWRKY10* was divided into six fragments (F1–F6). The full‐length CDS and each of the six fragments was separately recombined into the pGBKT7 bait vector. The full‐length CDS of *MdTTG1*, *MdMYB10*, *MdbHLH3*, and *MdbHLH33* was recombined into the pGADT7 vector. The Y2HGold yeast strain (Clontech, China) harboring the recombinant pGADT7 and pGBKT7 vectors was grown on a selective medium lacking Trp and Leu (−T/−L) and lacking Trp, Leu, His, and Ade (−T/−L/−H/−A). Each colony was dissolved in 10 ml sterile water and then diluted to 10^−1^ to 10^−3^. At least three colonies per combination were tested. The primers used for gene cloning and vector constructs were listed in Table S3, Supporting Information.

### Pull‐Down Assay

The full‐length CDS of *MdWRKY10* was cloned into the pET‐32a (+) vector fused to a HIS tag. The full‐length CDS of *MdTTG1*, *MdMYB10*, *MdbHLH3*, and *MdbHLH33* was cloned into the pGEX‐4T‐1 vector fused to a GST tag. The recombinant vectors were transformed into *Escherichia coli* BL21 strain for prokaryotic protein induction with 0.5 mM isopropylthio‐β‐galactoside. The MdWRKY10‐His and MdTTG1‐GST fusion proteins were purified using corresponding affinity purification columns. The pull‐down assays were performed using a His‐tagged protein purification kit (Clontech) as previously described.^[^ ^62^ ^]^ The two proteins were mixed and added to a cobalt chelating affinity resin containing immobilized His‐tagged bait protein (Clontech), incubated at 4 °C for 8–12 h, and the pulled‐down protein was detected with GST antibody after elution. The primers used for gene cloning and vector constructs were listed in Table S3, Supporting Information.

### BiFC Assay

The BiFC assays were conducted as previously described.^[^ ^62^ ^]^ Full‐length CDSs of *MdWRKY10*, *MdTTG1*, *MdMYB10*, *MdbHLH3*, and *MdbHLH33* without stop codons were inserted into the pSPYCE‐35S and pSPYNE‐35S vectors, respectively. The recombinant vectors were transformed into *Agrobacterium tumefaciens* GV3101 strain. The transformed GV3101 strains containing *MdWRKY10* and *MdTTG1* were mixed and co‐infiltrated into leaves of 4–6‐week‐old *Nicotiana benthamiana* plants. After co‐infiltration for 48 h in the dark, the YFP signals were detected by epifluorescence microscopy at an excitation wavelength of 488 nm (NIKON ECLIPSE Ni‐E, Tokyo, Japan). The primers used for gene cloning and vector constructs were listed in Table S3, Supporting Information.

### Co‐IP Assay

The Co‐IP assays were conducted as previously described.^[^ ^62^ ^]^ Full‐length CDSs of *MdWRKY10* and *MdTTG1* without stop codons were cloned into the pRI101‐AN‐GFP and pCB302‐HA vectors, respectively. The recombinant *MdWRKY10*‐GFP and *MdTTG1*‐HA vectors were individually or jointly transformed into apple calli prepared for the Co‐IP assays. Total proteins were extracted from each transgenic calli and incubated with GFP antibody (Abmart, Shanghai, China) for 2 h before adding agarose beads (Sigma, Darmstadt, Germany). The beads were rinsed and the precipitate was analyzed by immunoblot analysis with the HA antibody. The primers used for gene cloning and vector constructs were listed in Table S3, Supporting Information.

### Genetic Transformation of Apple Calli

To generate *MdWRKY10*‐overexpression apple calli, the full‐length CDS of *MdWRKY10* without stop codons was cloned into the pRI101‐AN vector driven by the CaMV 35S promoter and harboring a GFP tag. The full‐length CDS of *MdTTG1* without stop codons was cloned into the pCB302 vector driven by the CaMV 35S promoter and harboring an HA tag. The recombinant vectors were transformed into *A. tumefaciens* strain LBA4404. After 20–30 min of infection, apple calli were transferred to MS solid medium and co‐cultured in the dark for 48 h. Then, the apple calli containing recombinant pRI101‐AN and pCB302 vectors were transferred to kanamycin and glufosinate screening medium, respectively.

To generate *MdWRKY10* or *MdTTG1* knockdown apple calli, a CRISPR/Cas9 gene editing toolkit was used as previously described.^[^ ^64^ ^]^ Two gRNAs were generated to target sites from the genome sequences of *MdWRKY10* and *MdTTG1* based on the PAM using an online tool (http://crispr.hzau.edu.cn/CRISPR2/). The designed gRNAs were reconstituted into the pKSE401 vector and then transformed into *A. tumefaciens* strain LBA4404. After infection, the apple calli containing recombinant pKSE401 vectors were transferred to kanamycin screening medium. The genomic DNAs of the screened positive transgenic calli were extracted for PCR amplification using gene‐specific primers. The PCR products were sequenced and compared, and the mutant transgenic lines with targeted editing were screened for further analysis. The gRNAs and the primers used for gene cloning and vector constructs were listed in Table S3, Supporting Information.

### Transient Expression of MdWRKY10 in Apple Fruit

Virus‐induced gene silencing was performed in apple fruit as previously described.^[^ ^21^ ^]^ A 402‐bp partial coding sequence specific to *MdWRKY10* was inserted into the pTRV2 virus vector and then introduced into *A. tumefaciens* GV3101 strain using the freeze‐thaw method. *A. tumefaciens* containing the recombinant pTRV2 vector and the auxiliary pTRV1 vector (mixed in a 1:1 ratio) were re‐suspended in infiltration buffer (10 mM MES, 10 mM MgCl_2_, and 150 µM acetosyringone, pH 5.5–5.7) and injected into “Fuji” apple fruit. After infiltration, the fruits were placed in an incubator at 24 °C under a 16/8 h (light/dark) photoperiod. After 3 d, the fruit injection sites were sampled for transgene verification and RT‐qPCR analysis.

For transient overexpression in fruit, the CDS of *MdWRKY10* was cloned and inserted into the pHB vector harboring the CaMV 35S promoter. The recombinant pHB vector was introduced into *A. tumefaciens* GV3101 strain. Subsequent injection and validation steps were the same as described for the virus‐induced gene silencing. The primers used for gene cloning and vector constructs were listed in Table S3, Supporting Information.

### RNA Extraction and Reverse Transcription‐Quantitative PCR (RT‐qPCR) Analysis

Total RNA was extracted from apple flesh and calli following the manufacturer's instructions using a polysaccharide and polyphenol RNA extraction kit (Tiangen, Beijing, China). After removing genomic DNA, total RNA was reverse‐transcribed into cDNA using a RevertAid first strand cDNA synthesis kit (Tiangen). RT‐qPCR analysis was carried out using SYBR Green PCR master mix (TransGen Biotech, Beijing, China) on an iCycler iQ5 system (BioRad, Hercules, CA, USA) as previously described.^[^ ^62^ ^]^ The total volume of the RT‐qPCR mixture was 20 µL, containing 10 µL of 2 *×* SYBR Green qPCR master Mix, 1 µL of forward and reverse primers (0.5 µM of each), and 100 ng of template cDNA. Relative gene transcript levels were calculated using the (Ct) 2^−ΔΔCt^ method.^[^ ^65^ ^]^ The primers used for RT‐qPCR were designed by Beacon Designer software and were listed in Table S3, Supporting Information.

### Genome Structural Variation (SV) Detection

Genomic DNA of red‐ and non‐red‐fleshed apples was isolated from apple leaf according to the SDS‐CTAB method. After the DNA was qualified, the library was constructed using ONT's ligation sequencing kit (SQK‐LSK109) and sequenced on an ONT PromethION machine. Base calling was performed using ONT Guppy v4.0.15 and quality was assessed with NanoPlot v1.31.0. Reads were aligned to reference genome GDDH13^[^ ^66^ ^]^ using minimap2 v2.17 and processed using samtools v1.9. SVs were called on both alignments with snifes v1.0.12 and SVIM v1.4.1. A minimum of two supporting reads were required to call an SV. The annotation of the SVs was conducted by ANNOVAR.

### GUS Expression and Staining of Transgenic Apple Calli

Different promoter sequences of *MdWRKY10* containing a 3‐repeat *R‐InD* (*3 × R‐InD::GUS*), single *R‐InD* (*R‐InD::GUS*), and *R‐InD*‐deficient (*Def::GUS*) sequence fused with a minimal 35S sequence were cloned and fused upstream of the GUS gene in the pBI121 vector. The empty promoter (*Empty::GUS*) and CaMV 35S promoter (*35S::GUS*) served as negative and positive controls, respectively. The genetic transformation for apple calli was performed as above. The expression of GUS was detected by RT‐qPCR analysis and histochemical staining assays as described previously.^[^ ^67^ ^]^ The primers used for promoter cloning, vector constructs and RT‐qPCR were listed in Table S3, Supporting Information.

### ChIP‐Seq

The stable transgenic apple calli overexpressing *MdWRKY10* fused to a GFP tag were prepared for the ChIP assay. The ChIP assays were performed following the manufacturer's instructions of the EZ‐ChIP 244 chromatin immunoprecipitation kit (Upstate, Waltham, MA, USA) as previously described.^[^ ^62^ ^]^ The apple calli expressing an empty GFP tag was used as a negative control. ChIP DNA degradation and contamination were monitored on agarose gels. The purified DNA fragments of the *MdWRKY10* immunoprecipitation and the input with two biological replicates were extracted to construct the libraries. The library preparations were sequenced on an Illumina HiSeq platform (Illumina, CA, USA) and 50‐bp single‐end reads or 150‐bp paired‐end reads were generated. Clean reads were obtained by removing reads containing adapters or poly‐N, and low quality reads from the raw data. After mapping reads to the reference genome, the MACS2 (version 2.1.0) was used^[^ ^68^ ^]^ peak calling software to identify regions of IP enrichment over the background. A q‐value threshold of 0.05 was used for all data sets. Statistical enrichment of the peak‐related genes in KEGG pathways (http://www.genome.jp/kegg/) was analyzed. DREME and HOMER software suites were used to detect de novo sequence motifs and match known motifs.

### Y1H Assay

The Y1H assays were conducted as previously described.^[^ ^62^ ^]^ The full‐length CDS of *MdWRKY10* was cloned into the pGADT7 vector. The promoter sequences of *MdWRKY10* containing *R‐InD*, *MdTTG1*, *MdMYB10*, *MdbHLH3*, *MdbHLH33*, *MdF3’5’H*, and *MdUFGT* were cloned and incorporated into the pHIS2 vector. The self‐activation of the background histidine leakiness in the pHIS2 vector was suppressed using 3‐amino‐1,2,4‐triazole (3‐AT). The Y187 yeast strain harboring the recombinant pGADT7 and pHIS2 vectors was grown on a selective medium lacking Trp, His, and Leu (−T/−H/−L) with the optimal concentrations of 3‐AT to determine the combinations. The primers used for vector constructs were listed in Table S3, Supporting Information.

### EMSA

The full‐length CDS of *MdWRKY10* was cloned into the pET‐32a (+) vector fused to a HIS tag and the protein was then induced and purified as described for the pull‐down assay. The EMSAs were performed following the manufacturer's instructions using the LightShift chemiluminescent EMSA kit (Thermo Scientific, Rockford, IL, USA). The 3′ biotin end‐labeled promoter sequences containing W‐boxes were listed in Table S6, Supporting Information. Unlabeled probes were used as competitors. The mutant probes were labeled and contained two or six mutated nucleotides.

### Luciferase (LUC) Assay

The full‐length CDS of *MdWRKY10* was inserted into the pGreenII 62‐SK vector as an effector. The promoter sequences of *MdWRKY10* containing *3 × R‐InD*, *R‐InD*, and deficient *R‐InD*, and *MdMYB10*, *MdF3’5’H* and *MdUFGT* were cloned and incorporated into the pGreenII 0800‐LUC vectors as reporters. The recombinant vectors were integrated into *A. tumefaciens* GV3101 strain. Both *A. tumefaciens* cultures expressing *MdWRKY10* and promoters were mixed and co‐infiltrated into 4–6‐week‐old *N. benthamiana* leaves. After co‐infiltration for 48 h in the dark, the LUC activity and luminescence intensity was detected using a living imaging analysis system (NightOWL II LB983, Berthold, Germany). The primers used for vector constructs were listed in Table S3, Supporting Information.

### Statistical Analysis

All experimental data were analyzed according to one‐way analysis of variance (ANOVA) followed by Tukey's multiple range test with SPSS Statistics 22 (IBM Corporation, NY, USA). Differences were considered significant for *P < 0.05; **P < 0.01; and ***P < 0.001. GraphPad Prism 8.0, Microsoft Excel 2016, and IBM SPSS Statistics 22 were used for analysis and mapping. Statistical data were provided in Table S7, Supporting Information.

### Accession Numbers

Sequence data from this article can be found in the Genome Database for Rosaceae (https://www.rosaceae.org/) or GenBank/EMBL libraries under accession numbers MdWRKY10, MD09G1056600; MdMYB10, MD09G1278600; MdbHLH3, MD11G1286900; MdbHLH33, MD07G1137500; MdTTG1, MD01G1228700; MdWRKY1, MD09G1121600; MdWRKY2, MD03G1044400; MdWRKY3, MD16G1066500; MdWRKY20, MD03G1188900; MdWRKY26, MD11G1059400; MdWRKY32, MD02G1007900; MdWRKY33, MD12G1181000; MdWRKY44, MD12G1128800; MdActin, MD12G1140800; PhPH3, AMR43368.1; NtWRKY44, XP_0 097 73393; VvWRKY44, XP_0 022 75978.1; ZjWRKY44, XP_01 589 9555.2; FaWRKY44, XP_0 043 02832; PpWRKY44, XP_0 072 05137.1; PbWRKY26, XP_0 093 42023.2; FaWRKY3, XP_0 043 04267.1; PpWRKY4, XP_0 072 15541; PbWRKY3, XP_0 093 60587.2; VvWRKY4, XP_01 066 1104.1; ZjWRKY3, XP_04 832 7518.1; NtWRKY4, NP_0 013 12319.1.

## Conflict of Interest

The authors declare no conflict of interest.

## Author Contributions

N.W., W.L. contributed equally to this work. X.C., L.C., N.W., W.L., and Z.M. designed and supervised the experiment. N.W., W.J., and Z.M. carried out gene expression analysis and functional validation. S.Z., Q.Z., L.Y., H.W., H.F., Z.Z., Z.C., S.W. conducted sample collection, field experiments and phenotyping. N.W. and X.C. developed the genetic populations. N.W. and L.C. plotted manuscript figures. N.W., L.C., W.L., X.C. wrote the manuscript with inputs from all the other authors.

## Supporting information

Supporting Information

Supporting Information

Supporting Information

Supporting Information

Supporting Information

Supporting Information

Supporting Information

Supporting Information

Supporting Information

Supporting Information

Supporting Information

Supporting Information

Supporting Information

Supporting Information

Supporting Information

Supporting Information

Supporting Information

Supporting Information

Supporting Information

Supplementary Table S1

Supplementary Table S2

Supplementary Table S3

Supplementary Table S4

Supplementary Table S5

Supplementary Table S6

Supplementary Table S7

## Data Availability

The data that support the findings of this study are available in the supplementary material of this article.
