# Supplementary material for: A Functional InDel in the WRKY10 Promoter Controls the Degree of Flesh Red Pigmentation in Apple
Source: Adv Sci (Weinh). 2024 Jun 14;11(30):2400998. doi: 10.1002/advs.202400998 (PMC11321683; doi:10.1002/advs.202400998)
Supplement: Supplementary file 1 — Supporting Information [file ADVS-11-2400998-s022.pdf]

ADVANCED SCIENCE
Open Access

Supporting Information

for *Adv. Sci.*, DOI 10.1002/advs.202400998

A Functional InDel in the WRKY10 Promoter Controls the Degree of Flesh Red Pigmentation in Apple

Nan Wang, Wenjun Liu, Zhuoxin Mei, Shuhui Zhang, Qi Zou, Lei Yu, Shenghui Jiang, Hongcheng Fang, Zongying Zhang, Zijing Chen, Shujing Wu, Lailiang Cheng and Xuesen Chen**

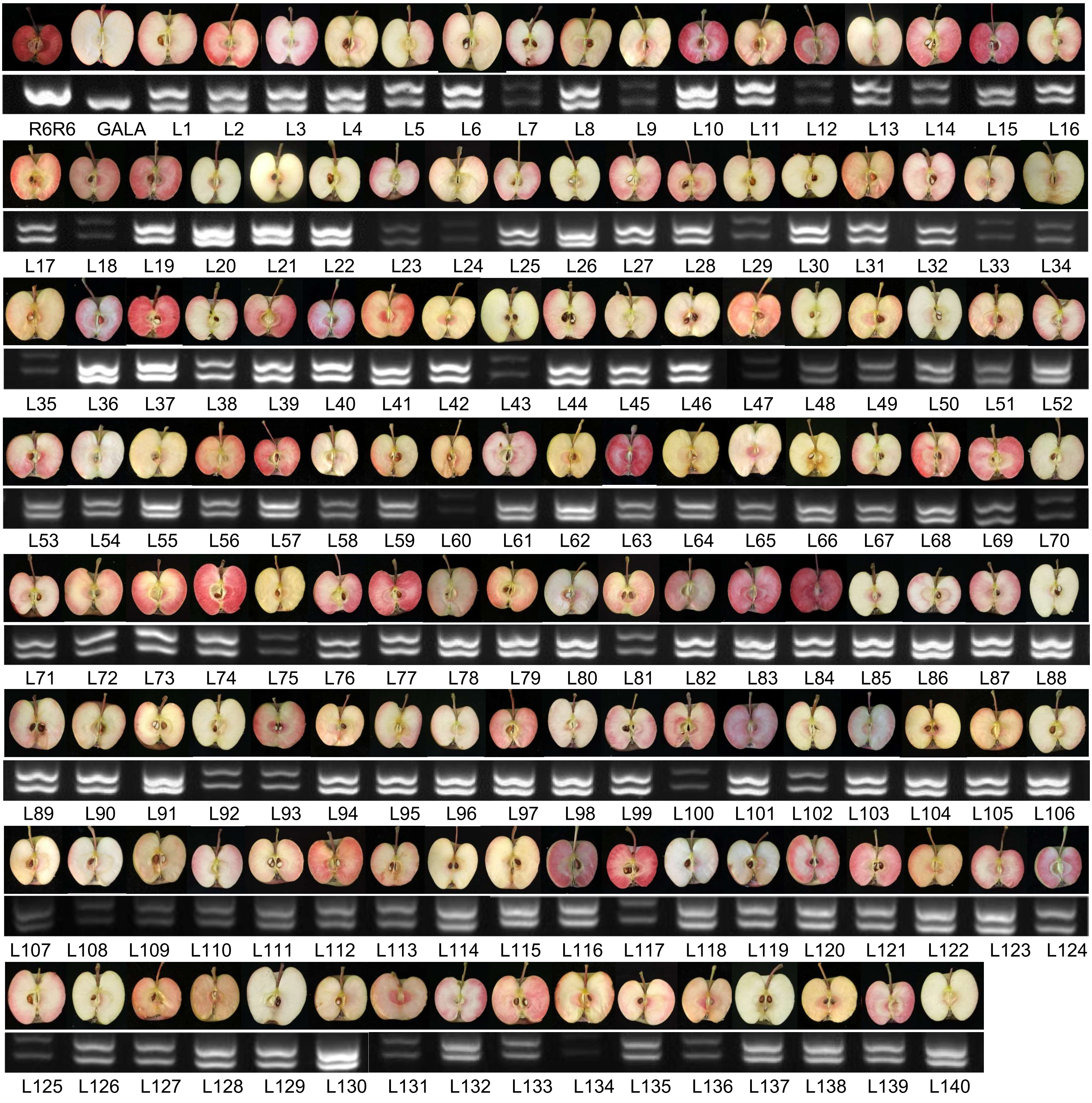

Supplemental Figure S1. The MdMYB10 promoter genotype in F_1 hybrid population. The R_6 and/or R_1 repetitive sequences were amplified by the primers designed from the *MdMYB10* promoter as described previously (Wang et al., 2017). Genotypes were then identified by agarose gel electrophoresis. R6R6: a previously identified R6:*MdMYB10* homozygous for the R6R6 genotype as the male parent. GALA: cultivated apple variety ‘Royal Gala’, as female parent.
