# Supplementary material for: A Functional InDel in the WRKY10 Promoter Controls the Degree of Flesh Red Pigmentation in Apple
Source: Adv Sci (Weinh). 2024 Jun 14;11(30):2400998. doi: 10.1002/advs.202400998 (PMC11321683; doi:10.1002/advs.202400998)
Supplement: Supplementary file 4 — Supporting Information [file ADVS-11-2400998-s020.pdf]

ADVANCED SCIENCE
Open Access

Supporting Information

for *Adv. Sci.*, DOI 10.1002/advs.202400998

A Functional InDel in the WRKY10 Promoter Controls the Degree of Flesh Red Pigmentation in Apple

Nan Wang, Wenjun Liu, Zhuoxin Mei, Shuhui Zhang, Qi Zou, Lei Yu, Shenghui Jiang, Hongcheng Fang, Zongying Zhang, Zijing Chen, Shujing Wu, Lailiang Cheng and Xuesen Chen**

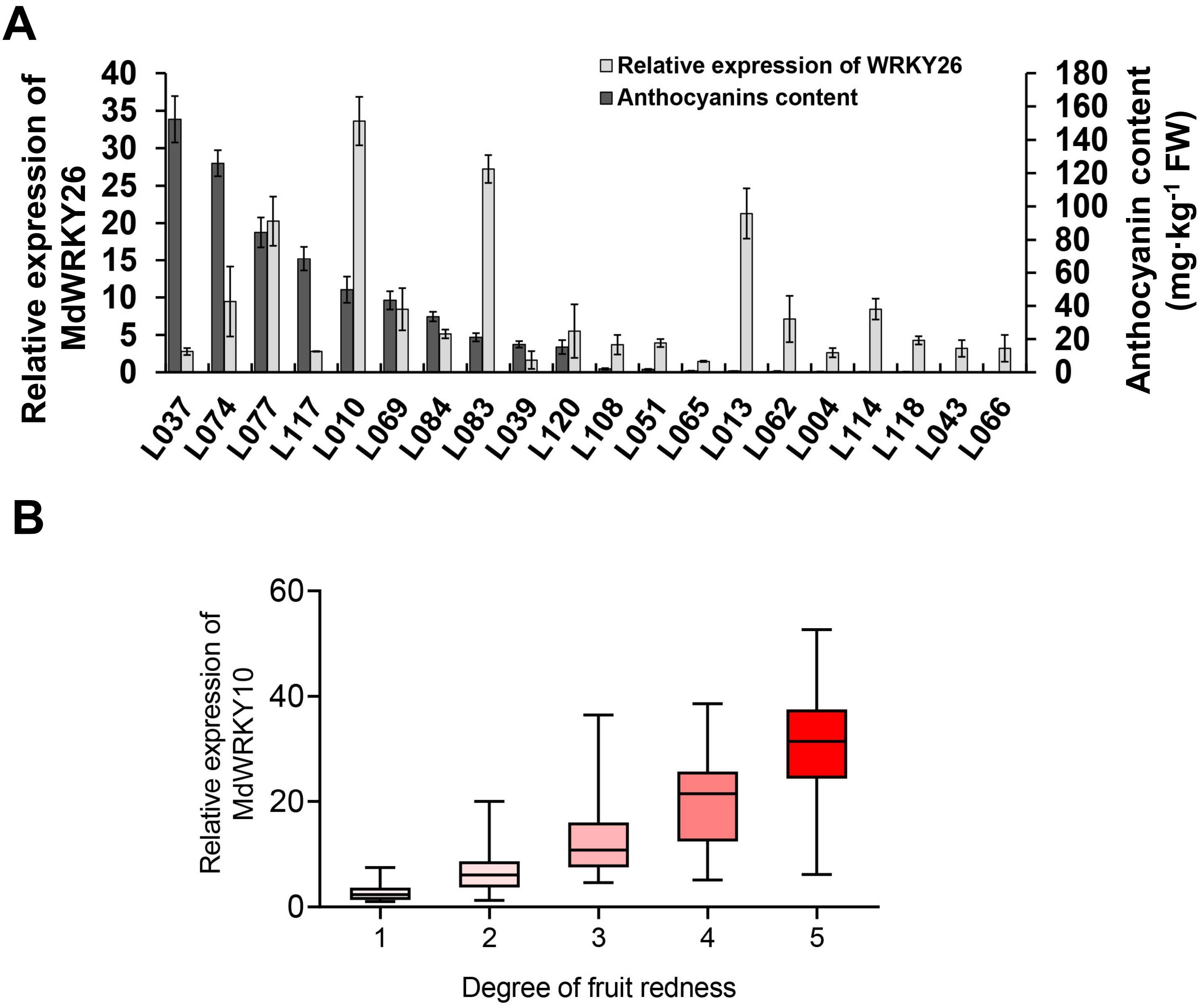

Supplemental Figure S4. The relationship of MdWRKY10, MdWRKY26 and the degree of flesh red coloration. (a) The content of anthocyanins and the transcript levels of MdWRKY26 in the selected 10 red-fleshed apple lines and 10 white-fleshed apple lines. FW: fresh wight. (b) The redness of fruit flesh in all progenies were categorized into 5 groups based on color values measured by a colorimeter, and the transcript levels of MdWRKY10 were detected in each group. Values are means ± SD of three independent biological replicates (n = 3).
