# Supplementary material for: A Functional InDel in the WRKY10 Promoter Controls the Degree of Flesh Red Pigmentation in Apple
Source: Adv Sci (Weinh). 2024 Jun 14;11(30):2400998. doi: 10.1002/advs.202400998 (PMC11321683; doi:10.1002/advs.202400998)
Supplement: Supplementary file 6 — Supporting Information [file ADVS-11-2400998-s016.pdf]

ADVANCED SCIENCE
Open Access

Supporting Information

for *Adv. Sci.*, DOI 10.1002/advs.202400998

A Functional InDel in the WRKY10 Promoter Controls the Degree of Flesh Red Pigmentation in Apple

Nan Wang, Wenjun Liu, Zhuoxin Mei, Shuhui Zhang, Qi Zou, Lei Yu, Shenghui Jiang, Hongcheng Fang, Zongying Zhang, Zijing Chen, Shujing Wu, Lailiang Cheng and Xuesen Chen**

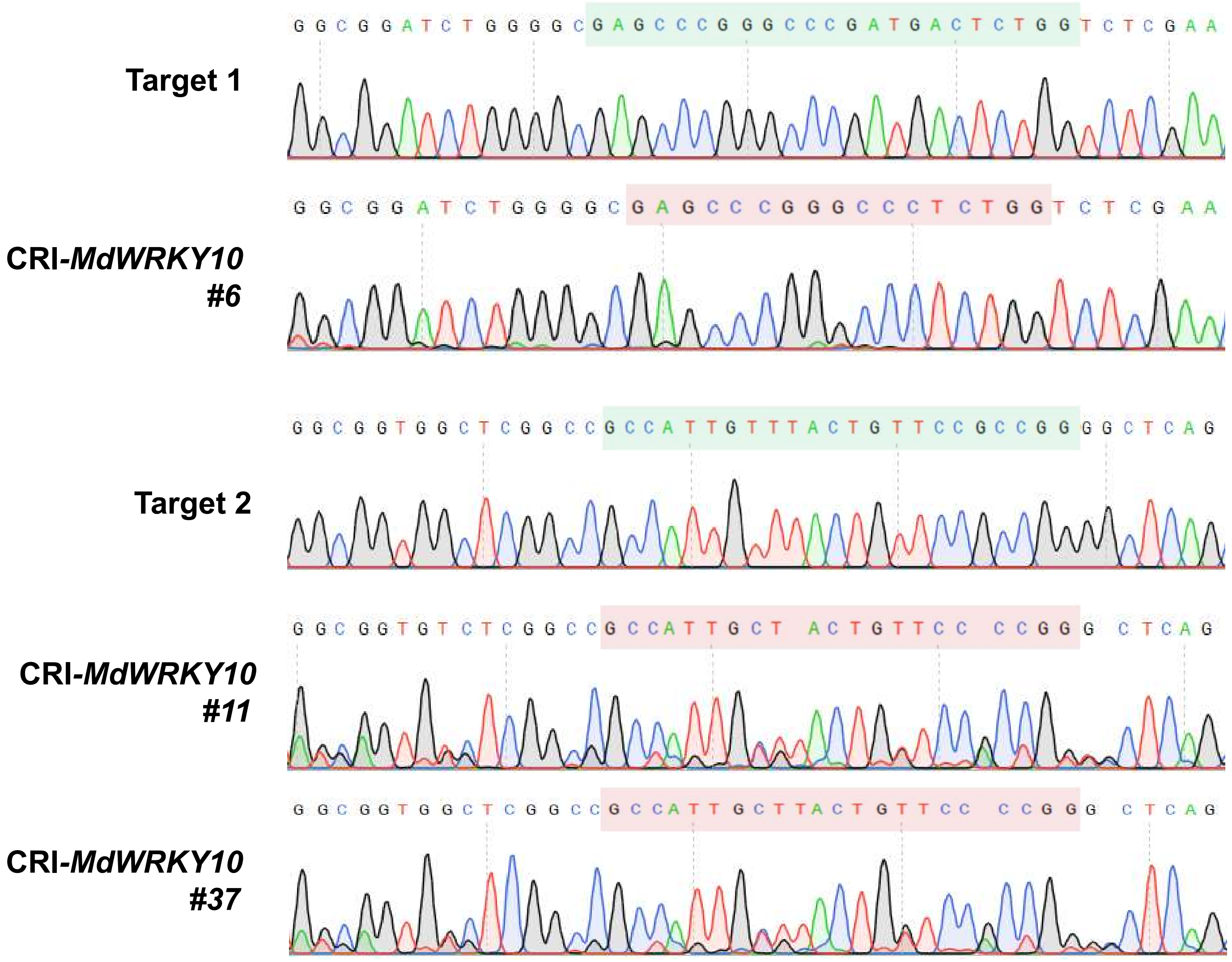

Supplemental Figure S6. PCR and sequencing results of three independent MdWRKY10 knockdown lines by CRISPR/cas9. Two gRNAs were designed as target sites (Target 1 and Target 2) from the first exon sequence of *MdWRKY10* based on the PAM. The knockdown lines displayed mutation and/or deletion sequences in the MdWRKY10 gene, including #6 in Target 1, #11 and #37 in Target 2. gRNA targeted sequences were labeled by green background, and the mutation or deletion sequences were labeled by red background.
