# Supplementary material for: A Functional InDel in the WRKY10 Promoter Controls the Degree of Flesh Red Pigmentation in Apple
Source: Adv Sci (Weinh). 2024 Jun 14;11(30):2400998. doi: 10.1002/advs.202400998 (PMC11321683; doi:10.1002/advs.202400998)
Supplement: Supplementary file 7 — Supporting Information [file ADVS-11-2400998-s010.pdf]

ADVANCED SCIENCE
Open Access

Supporting Information

for *Adv. Sci.*, DOI 10.1002/advs.202400998

A Functional InDel in the WRKY10 Promoter Controls the Degree of Flesh Red Pigmentation in Apple

Nan Wang, Wenjun Liu, Zhuoxin Mei, Shuhui Zhang, Qi Zou, Lei Yu, Shenghui Jiang, Hongcheng Fang, Zongying Zhang, Zijing Chen, Shujing Wu, Lailiang Cheng and Xuesen Chen**

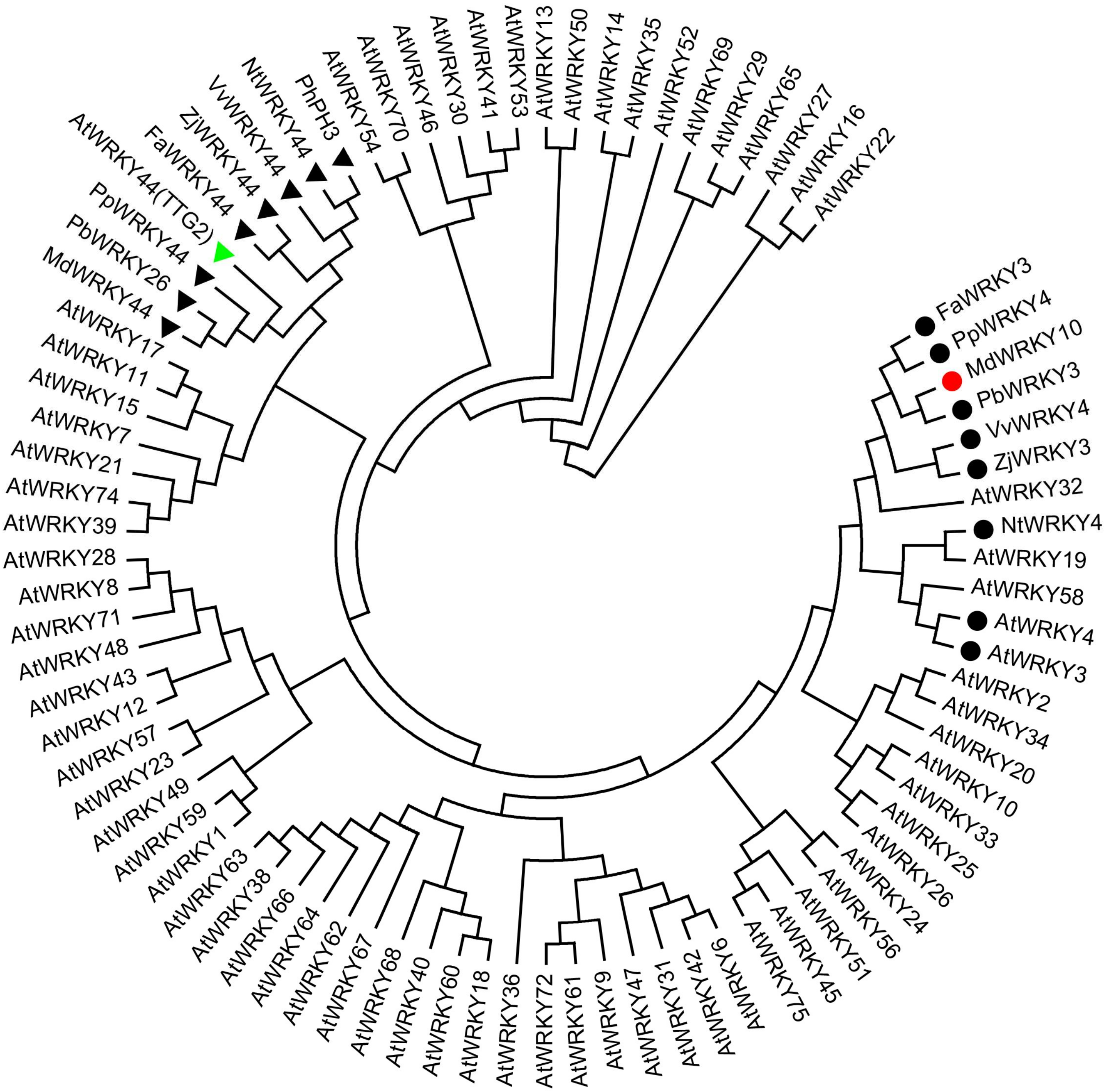

Supplemental Figure S7. Phylogenetic analysis of the homologous proteins of MdWRKY10 and TTG2 from additional. Black dots indicate homologous proteins of MdWRKY10, black triangles indicate the homologous proteins of TTG2; MdWRKY44:XP_008387690.2; PhPH3:AMR43368.1; NtWRKY44:XP_009773393; VvWRKY44: XP_002275978.1; ZjWRKY44: XP_015899555.2; FaWRKY44:XP_004302832.1; PpWRKY44: XP_007205137.1; PbWRKY26: XP_009342023.2; FaWRKY3: XP_004304267.1; PpWRKY4: XP_007215541.1; PbWRKY3: XP_009360587.2; VvWRKY4: XP_010661104.1; ZjWRKY3:XP_048327518.1; NtWRKY4: NP_001312319.1; The 71 *Arabidopsis thaliana* WRKY protein sequences were obtained from the TAIR database (http://www.arabidopsis.org/).
