# Supplementary material for: A Functional InDel in the WRKY10 Promoter Controls the Degree of Flesh Red Pigmentation in Apple
Source: Adv Sci (Weinh). 2024 Jun 14;11(30):2400998. doi: 10.1002/advs.202400998 (PMC11321683; doi:10.1002/advs.202400998)
Supplement: Supplementary file 8 — Supporting Information [file ADVS-11-2400998-s007.pdf]

ADVANCED SCIENCE
Open Access

Supporting Information

for *Adv. Sci.*, DOI 10.1002/advs.202400998

A Functional InDel in the WRKY10 Promoter Controls the Degree of Flesh Red Pigmentation in Apple

Nan Wang, Wenjun Liu, Zhuoxin Mei, Shuhui Zhang, Qi Zou, Lei Yu, Shenghui Jiang, Hongcheng Fang, Zongying Zhang, Zijing Chen, Shujing Wu, Lailiang Cheng and Xuesen Chen**

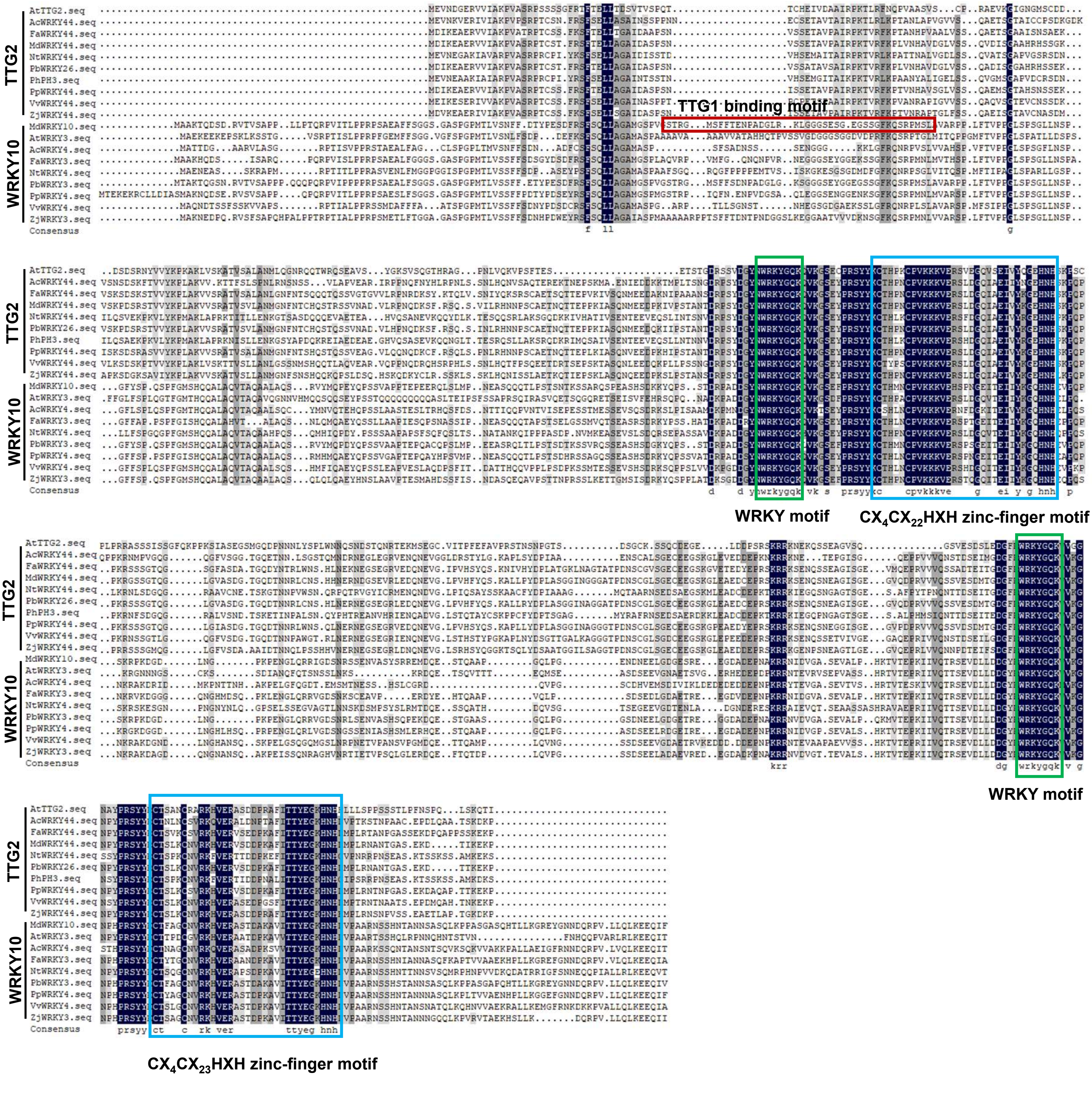

Supplemental Figure S8. Amino acid sequence alignment analysis of the MdWRKY10 and TTG2 homologous proteins. Both MdWRKY10 and TTG2 homologous proteins belong to the Subgroup I, which all members contained two WRKY domains each followed by a C2H2 zinc finger motif 16 amino acids behind. Red box represents the binding domain of MdWRKY10 to MdTTG1; Green boxes represents the conserved WRKY motif; Blue boxes represents the C2H2 zinc finger motif.
