# Supplementary material for: A Functional InDel in the WRKY10 Promoter Controls the Degree of Flesh Red Pigmentation in Apple
Source: Adv Sci (Weinh). 2024 Jun 14;11(30):2400998. doi: 10.1002/advs.202400998 (PMC11321683; doi:10.1002/advs.202400998)
Supplement: Supplementary file 9 — Supporting Information [file ADVS-11-2400998-s012.pdf]

ADVANCED SCIENCE Open Access

Supporting Information

for *Adv. Sci.*, DOI 10.1002/advs.202400998

A Functional InDel in the WRKY10 Promoter Controls the Degree of Flesh Red Pigmentation in Apple

Nan Wang, Wenjun Liu, Zhuoxin Mei, Shuhui Zhang, Qi Zou, Lei Yu, Shenghui Jiang, Hongcheng Fang, Zongying Zhang, Zijing Chen, Shujing Wu, Lailiang Cheng and Xuesen Chen**

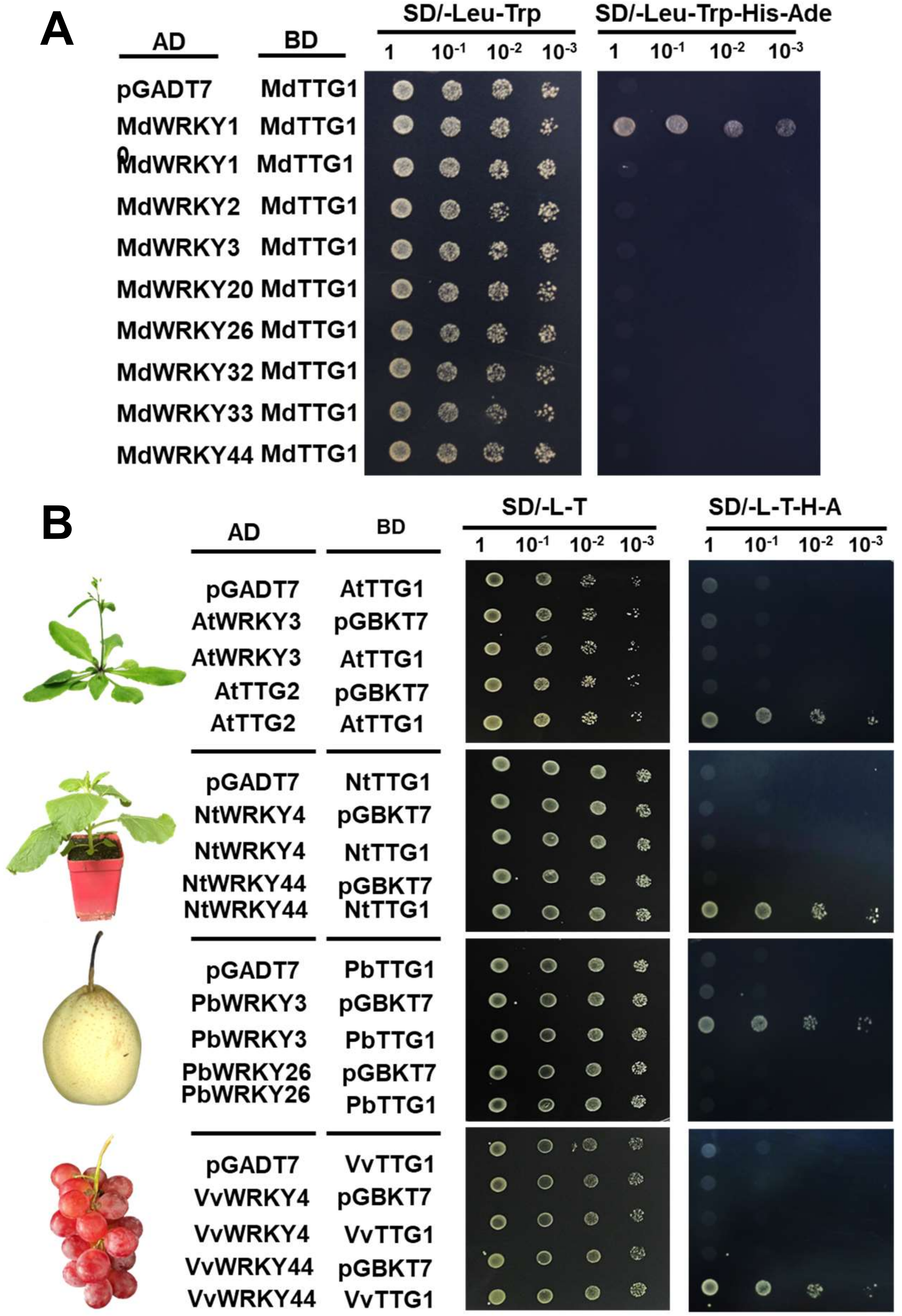

Supplemental Figure S9. The interaction between TTG1 and homologous genes of both TTG2 and WRKY10 clusters in apple and other different species. (a) Y2H assays showing the interaction of 8 WRKY TFs with MdTTG1. All of them, including the TTG2 homolog MdWRKY44, did not interacted with MdTTG1. The MdWRKY10-AD and MdTTG1-BD pair was used as a positive control. (b) Y2H assays showing the interaction between TTG1 and the homologous genes of both TTG2 and WRKY10 clusters in different species. Each colony was dissolved in 10 ml sterile water and then diluted to 10^{-1} to 10^{-3}. At least three colonies per combination were tested.
