# Supplementary material for: A Functional InDel in the WRKY10 Promoter Controls the Degree of Flesh Red Pigmentation in Apple
Source: Adv Sci (Weinh). 2024 Jun 14;11(30):2400998. doi: 10.1002/advs.202400998 (PMC11321683; doi:10.1002/advs.202400998)
Supplement: Supplementary file 10 — Supporting Information [file ADVS-11-2400998-s015.pdf]

ADVANCED SCIENCE Open Access

Supporting Information

for *Adv. Sci.*, DOI 10.1002/advs.202400998

A Functional InDel in the WRKY10 Promoter Controls the Degree of Flesh Red Pigmentation in Apple

Nan Wang, Wenjun Liu, Zhuoxin Mei, Shuhui Zhang, Qi Zou, Lei Yu, Shenghui Jiang, Hongcheng Fang, Zongying Zhang, Zijing Chen, Shujing Wu, Lailiang Cheng and Xuesen Chen**

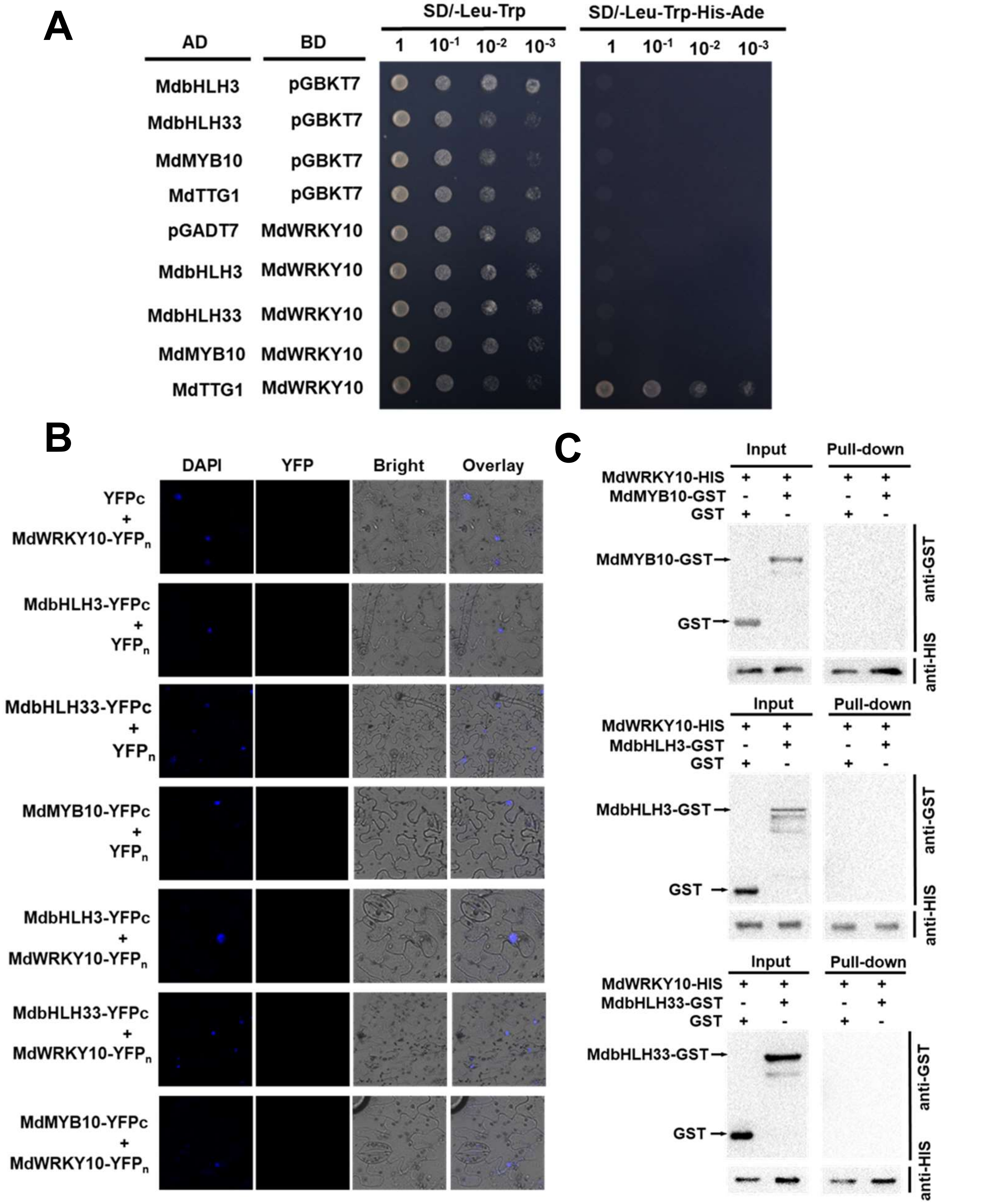

Supplemental Figure S10. The interaction between MdWRKY10 and other MBW complex members. (a) Y2H assays showing the interaction of MdWRKY10 with MdMYB10, MdbHLH3, and MdbHLH33. The MdTTG1-AD and MdWRKY10-BD pair was used as a positive control. All of them did not interacted with MdWRKY10. Each colony was dissolved in 10 ml sterile water and then diluted to 10^{-1} to 10^{-3}. At least three colonies per combination were tested. (b) BiFC assay showing the interaction of MdWRKY10 with MdMYB10, MdbHLH3, and MdbHLH33; DAPI staining was used to visualize the nuclei. (c) Pull-down assay showing the interaction of MdWRKY10 with MdMYB10, MdbHLH3, and MdbHLH33.
