# Supplementary material for: A Functional InDel in the WRKY10 Promoter Controls the Degree of Flesh Red Pigmentation in Apple
Source: Adv Sci (Weinh). 2024 Jun 14;11(30):2400998. doi: 10.1002/advs.202400998 (PMC11321683; doi:10.1002/advs.202400998)
Supplement: Supplementary file 11 — Supporting Information [file ADVS-11-2400998-s006.pdf]

ADVANCED SCIENCE
Open Access

Supporting Information

for *Adv. Sci.*, DOI 10.1002/advs.202400998

A Functional InDel in the WRKY10 Promoter Controls the Degree of Flesh Red Pigmentation in Apple

Nan Wang, Wenjun Liu, Zhuoxin Mei, Shuhui Zhang, Qi Zou, Lei Yu, Shenghui Jiang, Hongcheng Fang, Zongying Zhang, Zijing Chen, Shujing Wu, Lailiang Cheng and Xuesen Chen**

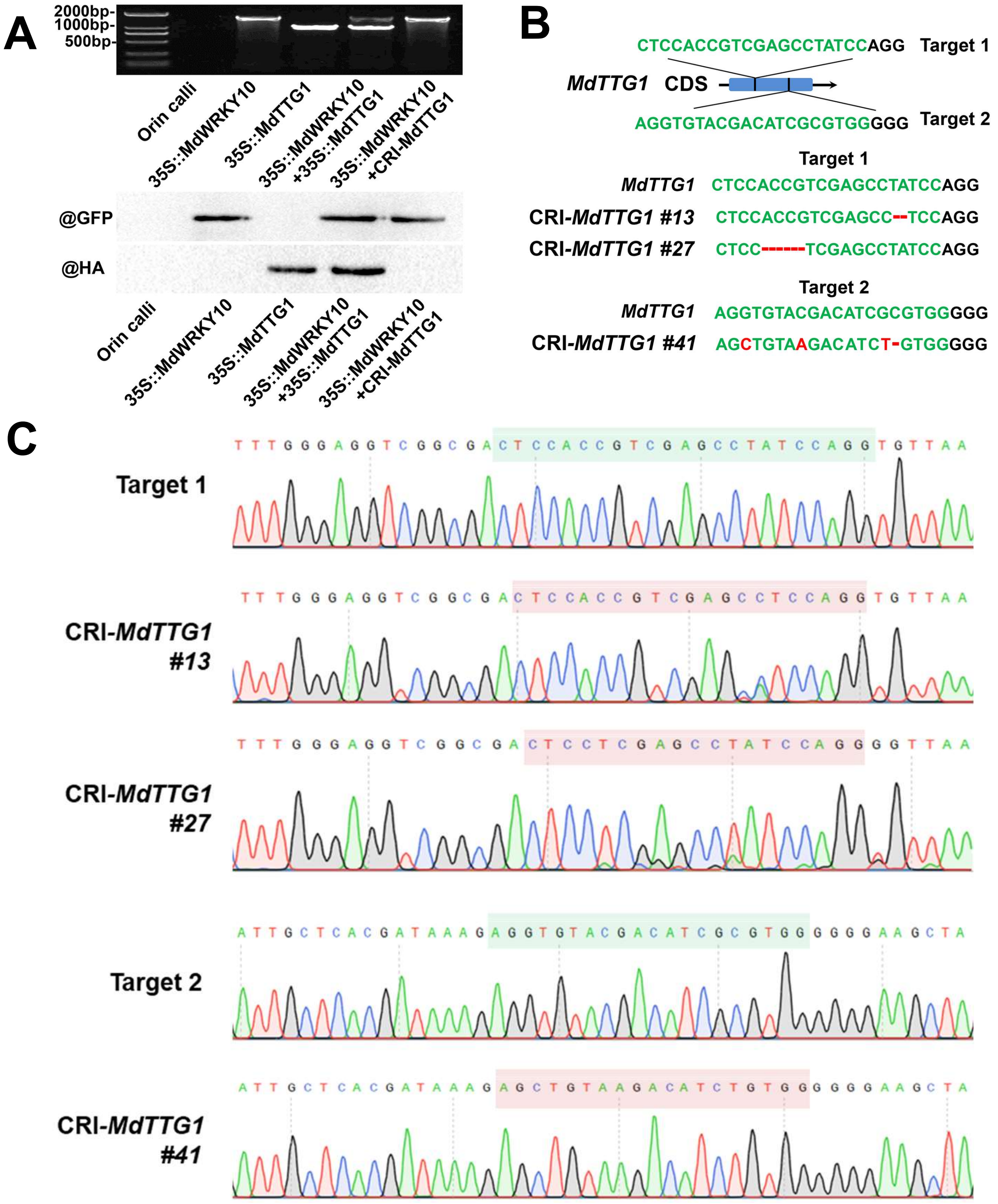

Supplemental Figure S11. PCR, immunoblotting, and sequencing results of overexpression and knockdown lines. (a) Presence of transgenes in *35S::MdWRKY10*, *35S::MdTTG1*, and *35S::MdWRKY10+35S::MdTTG1* calli were confirmed by PCR amplification and immunoblotting with GFP or HA antibody. (b) *MdTTG1*-knockdown lines #13, #27, and #41 were generated via CRISPR-Cas9 gene editing on a background of *35S::MdWRKY10* overexpression calli. (c) Two gRNAs were designed as target sites (Target 1 and Target 2) from the exon sequence of *MdTTG1* based on the PAM. The knockdown lines displayed mutation and/or deletion sequences in the MdTTG1 gene, including #13 and #27 in Target 1, and #41 in Target 2. gRNA targeted sequences were labeled by green background, and the mutation or deletion sequences were labeled by red background.
