# Supplementary material for: A Functional InDel in the WRKY10 Promoter Controls the Degree of Flesh Red Pigmentation in Apple
Source: Adv Sci (Weinh). 2024 Jun 14;11(30):2400998. doi: 10.1002/advs.202400998 (PMC11321683; doi:10.1002/advs.202400998)
Supplement: Supplementary file 12 — Supporting Information [file ADVS-11-2400998-s024.pdf]

ADVANCED SCIENCE
Open Access

Supporting Information

for *Adv. Sci.*, DOI 10.1002/advs.202400998

A Functional InDel in the WRKY10 Promoter Controls the Degree of Flesh Red Pigmentation in Apple

Nan Wang, Wenjun Liu, Zhuoxin Mei, Shuhui Zhang, Qi Zou, Lei Yu, Shenghui Jiang, Hongcheng Fang, Zongying Zhang, Zijing Chen, Shujing Wu, Lailiang Cheng and Xuesen Chen**

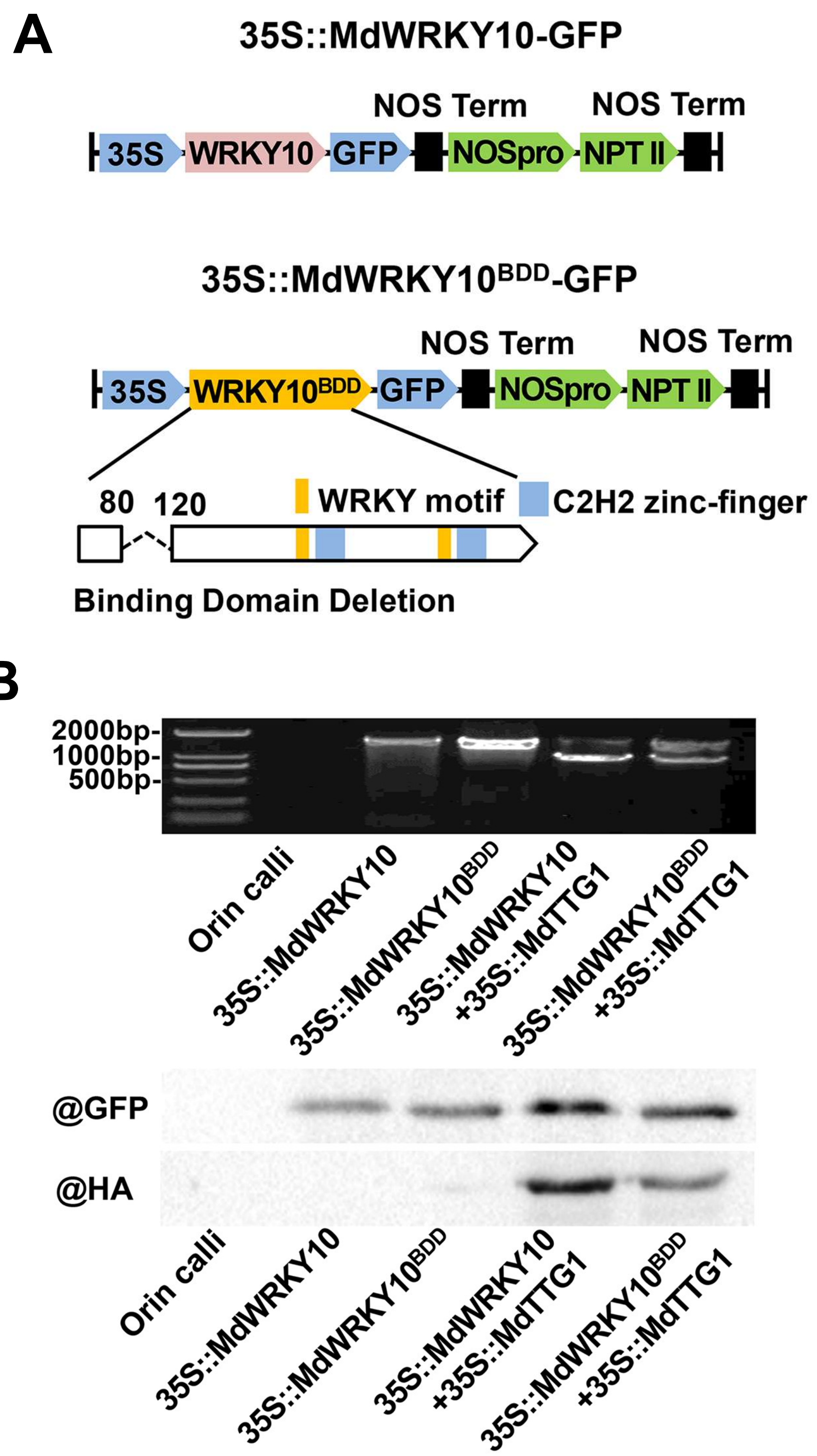

Supplemental Figure S12. Diagram of the recombinant vector and the verification of transgenic apple calli. (a) Diagram of the recombinant vector carrying full-length *MdWRKY10* (*35S::MdWRKY10*) and binding domain-deleted *MdWRKY10* (*35S::MdWRKY10*^{BDD}). (b) The presence of transgenes in *35S::MdWRKY10*, *35S::MdWRKY10*^{BDD}, *35S::MdWRKY10+35S::MdTTG1*, and *35S::MdWRKY10*^{BDD}*+35S::MdTTG1* calli were confirmed by PCR amplification and immunoblotting with GFP or HA antibody.
