# Supplementary material for: A Functional InDel in the WRKY10 Promoter Controls the Degree of Flesh Red Pigmentation in Apple
Source: Adv Sci (Weinh). 2024 Jun 14;11(30):2400998. doi: 10.1002/advs.202400998 (PMC11321683; doi:10.1002/advs.202400998)
Supplement: Supplementary file 13 — Supporting Information [file ADVS-11-2400998-s002.pdf]

ADVANCED SCIENCE
Open Access

Supporting Information

for *Adv. Sci.*, DOI 10.1002/advs.202400998

A Functional InDel in the WRKY10 Promoter Controls the Degree of Flesh Red Pigmentation in Apple

Nan Wang, Wenjun Liu, Zhuoxin Mei, Shuhui Zhang, Qi Zou, Lei Yu, Shenghui Jiang, Hongcheng Fang, Zongying Zhang, Zijing Chen, Shujing Wu, Lailiang Cheng and Xuesen Chen**

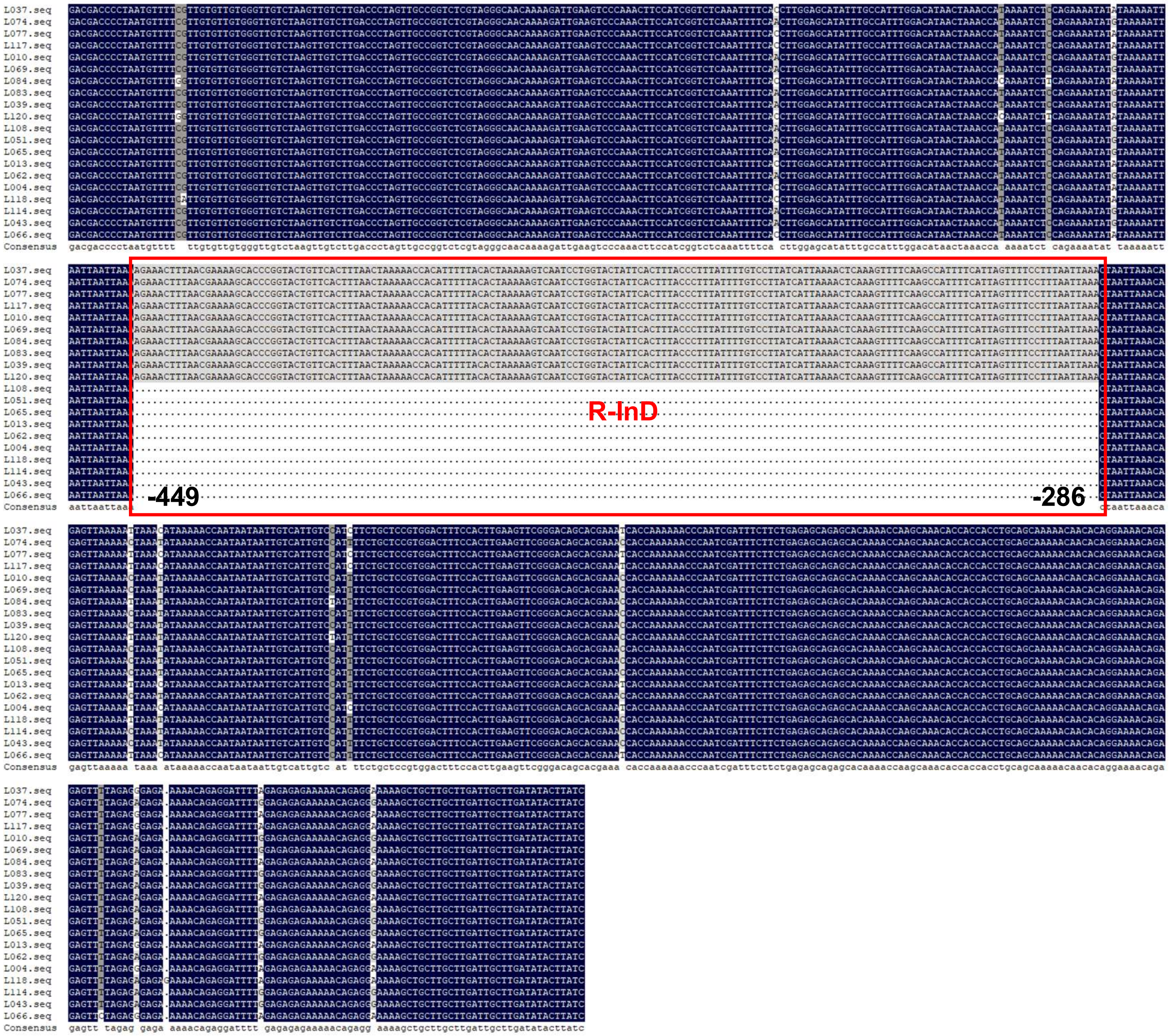

Supplemental Figure S13. Sequence alignment analysis of the *MdWRKY10* promoter between red- and white-fleshed apples. The promoter sequences of *MdWRKY10* used for alignment analysis were PCR amplification from genome DNA of selected 10 lines with the typical red flesh phenotype and 10 lines with the non-red flesh phenotype. Apple lines in the population were labeled as L001 to L140, as shown on the left. The long insertion sites in the promoter region (-449 – -286) of *MdWRKY10* are indicated by a red box (R-InD).
