# Supplementary material for: A Functional InDel in the WRKY10 Promoter Controls the Degree of Flesh Red Pigmentation in Apple
Source: Adv Sci (Weinh). 2024 Jun 14;11(30):2400998. doi: 10.1002/advs.202400998 (PMC11321683; doi:10.1002/advs.202400998)
Supplement: Supplementary file 14 — Supporting Information [file ADVS-11-2400998-s008.pdf]

ADVANCED SCIENCE Open Access

Supporting Information

for *Adv. Sci.*, DOI 10.1002/advs.202400998

A Functional InDel in the WRKY10 Promoter Controls the Degree of Flesh Red Pigmentation in Apple

Nan Wang, Wenjun Liu, Zhuoxin Mei, Shuhui Zhang, Qi Zou, Lei Yu, Shenghui Jiang, Hongcheng Fang, Zongying Zhang, Zijing Chen, Shujing Wu, Lailiang Cheng and Xuesen Chen**

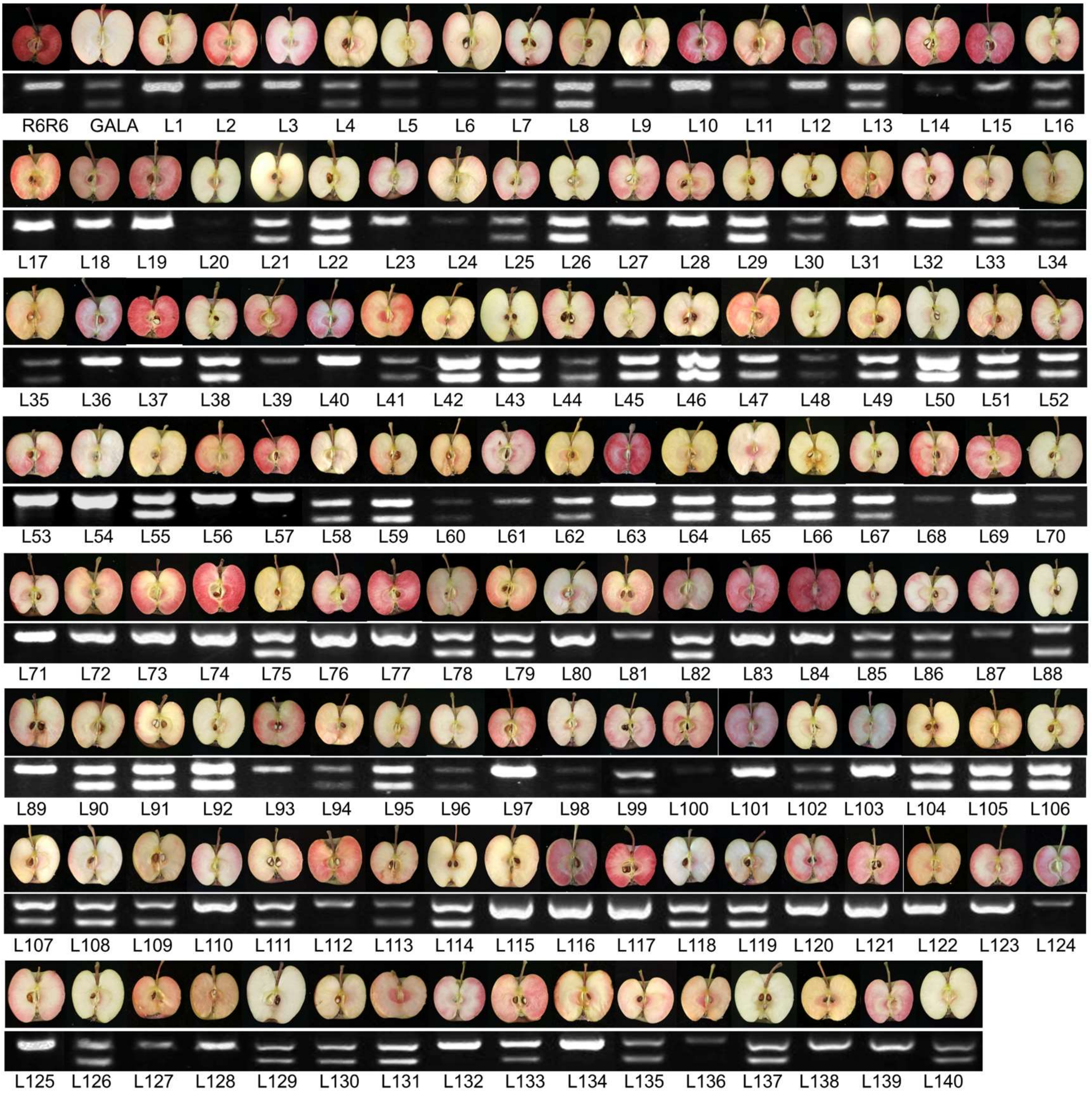

Supplemental Figure S14. The R-InD genotypes of MdWRKY10 promoter in F_1 hybrid population. The R-InD genotypes were identified by agarose gel electrophoresis from all 140 apple lines in the hybrid population. R6R6: a previously identified R6:*MdMYB10* homozygous for the R6R6 genotype as the male parent. GALA: cultivated apple variety ‘Royal Gala’, as female parent.
