# Supplementary material for: A Functional InDel in the WRKY10 Promoter Controls the Degree of Flesh Red Pigmentation in Apple
Source: Adv Sci (Weinh). 2024 Jun 14;11(30):2400998. doi: 10.1002/advs.202400998 (PMC11321683; doi:10.1002/advs.202400998)
Supplement: Supplementary file 16 — Supporting Information [file ADVS-11-2400998-s005.pdf]

ADVANCED SCIENCE
Open Access

Supporting Information

for *Adv. Sci.*, DOI 10.1002/advs.202400998

A Functional InDel in the WRKY10 Promoter Controls the Degree of Flesh Red Pigmentation in Apple

Nan Wang, Wenjun Liu, Zhuoxin Mei, Shuhui Zhang, Qi Zou, Lei Yu, Shenghui Jiang, Hongcheng Fang, Zongying Zhang, Zijing Chen, Shujing Wu, Lailiang Cheng and Xuesen Chen**

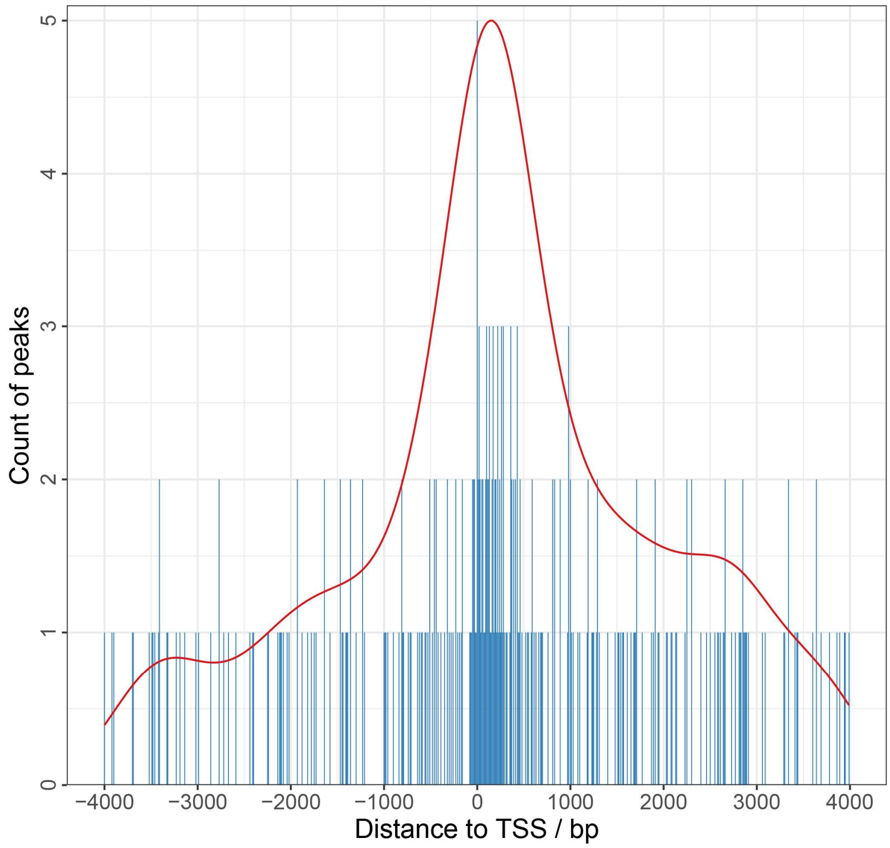

Supplemental Figure S16. The distribution of binding peaks at transcription start site (TSS) of related genes. The position of peak summit around TSS of genes can predict the interaction sites between MdWRKY10 and downstream genes. Abscissa: Distance from peak to TSS; Ordinate: Number of peaks.
