# Supplementary material for: A Functional InDel in the WRKY10 Promoter Controls the Degree of Flesh Red Pigmentation in Apple
Source: Adv Sci (Weinh). 2024 Jun 14;11(30):2400998. doi: 10.1002/advs.202400998 (PMC11321683; doi:10.1002/advs.202400998)
Supplement: Supplementary file 19 — Supporting Information [file ADVS-11-2400998-s026.pdf]

ADVANCED SCIENCE
Open Access

Supporting Information

for *Adv. Sci.*, DOI 10.1002/advs.202400998

A Functional InDel in the WRKY10 Promoter Controls the Degree of Flesh Red Pigmentation in Apple

Nan Wang, Wenjun Liu, Zhuoxin Mei, Shuhui Zhang, Qi Zou, Lei Yu, Shenghui Jiang, Hongcheng Fang, Zongying Zhang, Zijing Chen, Shujing Wu, Lailiang Cheng and Xuesen Chen**

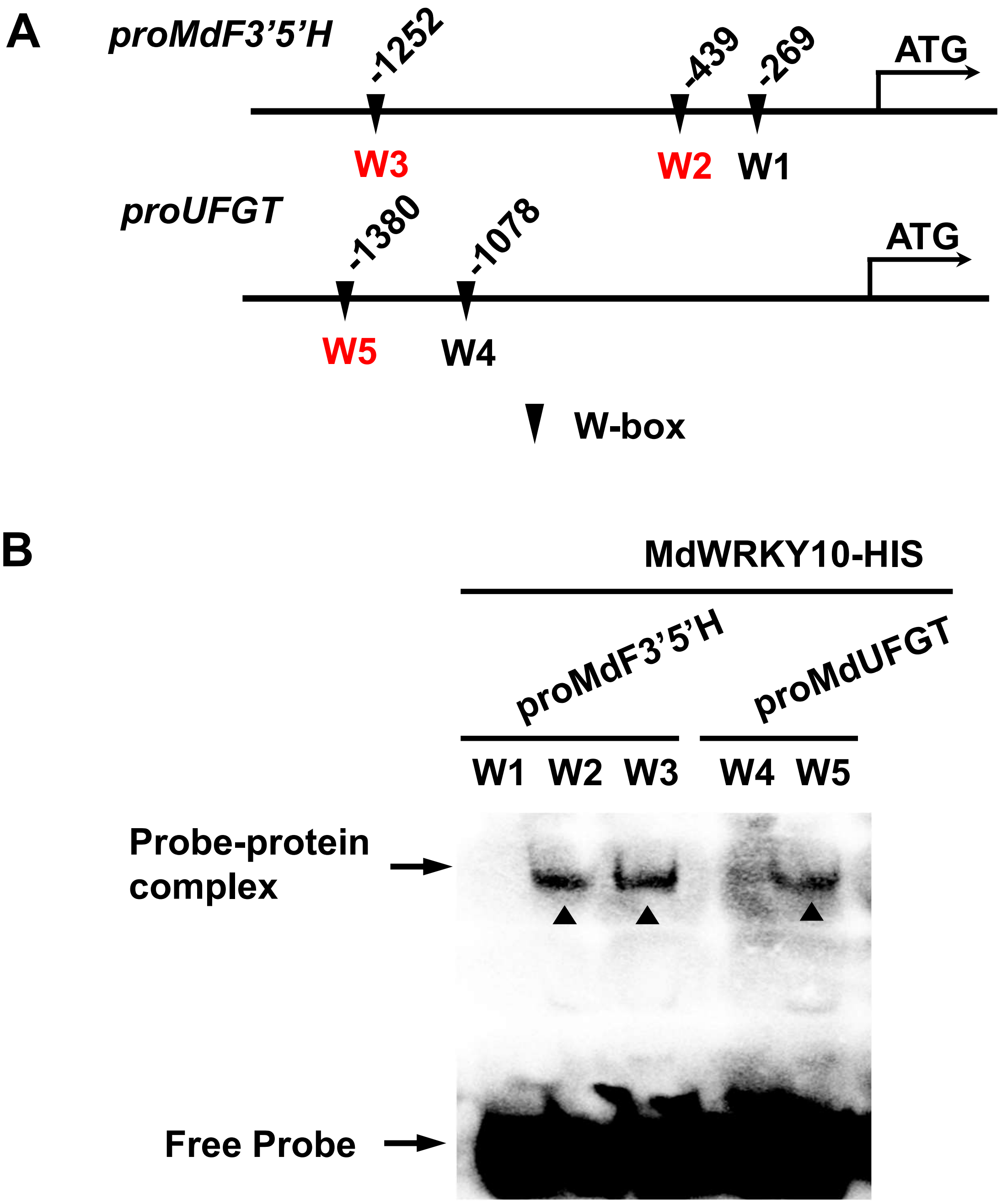

Supplemental Figure S19. EMSA showing binding of MdWRKY10 to the W-boxes in the promoters of *MdF3'5'H* and *MdUFGT*. (a) The W-box cis-elements in the promoters of *MdF3'5'H* and *MdUFGT.* Numbers indicate positions of the W-box cis-elements upstream of the coding sequence of *MdF3'5'H* and *MdUFGT.* (b) EMSA showing the binding of MdWRKY10 to the W-box cis-elements in the promoter of *MdF3'5'H* and *MdUFGT.* MdWRKY10-HIS: MdWRKY10 fused to a histidine (His) tag. The black triangles represented that MdWRKY10 combined with the W2, W3 and W5 cis-elements.
